# Lipid nanocarriers as innovative strategies for hair regrowth

**DOI:** 10.1186/s11671-026-04702-7

**Published:** 2026-06-30

**Authors:** Rabab Kamel, Shimaa K. Mohamed, Mona M. AbouSamra

**Affiliations:** 1 Pharmaceutical Technology Department, National Research Centre, Cairo, 12622 Egypt; 2 Department of Pharmacology and Toxicology, Faculty of Pharmacy, Capital University (formerly Helwan University), Cairo, Egypt

**Keywords:** Hair loss, Scalp, Lipid nanoparticles, Drug targeting, Dermatologic

## Abstract

Alopecia, or hair loss, is a multifactorial condition that may arise from genetic, hormonal, or autoimmune causes. This review outlines the major types of alopecia and discusses their underlying etiologies, along with the limitations associated with current therapeutic approaches, including poor efficacy, adverse effects, and low patient adherence. In recent years, lipid-based nanoparticles have emerged as promising carriers for improving the delivery of therapeutic agents to the scalp and hair follicles. The present review summarizes different treatment strategies for alopecia while highlighting the potential role of lipid-based nanocarriers in enhancing drug delivery. The review critically summarizes recent advances in the use of these nanocarriers for alopecia management, with focus on their ability to improve skin penetration, follicular targeting, drug stability, and sustained release characteristics. In addition, the advantages and limitations of different lipid-based systems are comparatively discussed, together with current challenges related to formulation stability, large-scale production, safety, and regulatory considerations in dermatological nanomedicine. Overall, this review provides an updated perspective on the therapeutic potential and translational prospects of lipid based nanocarriers as emerging platforms for alopecia treatment, while highlighting the need for further preclinical and clinical validation.

**Graphical abstract:**

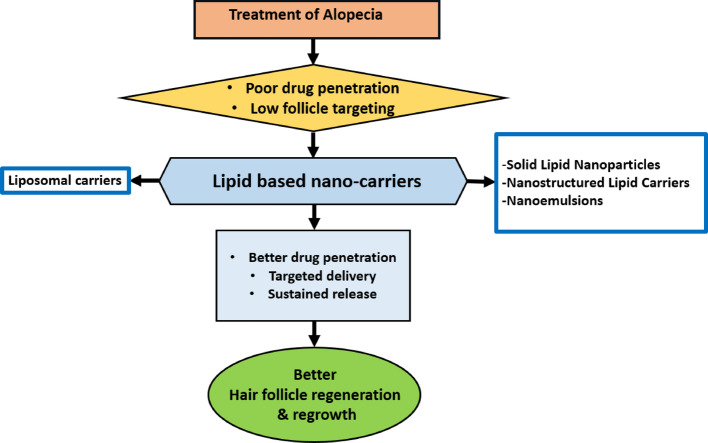

## Introduction

Alopecia is a disorder that leads to total loss of hair from the scalp or other parts of the body which are normally characterized by hair growth. Hair loss is a prevalent problem suffered by multitudes worldwide, regardless of age or gender. It can be a frustrating condition for numerous individuals, undermining their confidence and sense of self-worth [1, 2].

The human head loses about 50–125 hairs per day (depending on sex), but most of them grow back during the resting stage when the follicle is not abolished, but when loss exceeds regrowth challenges emerge. Pathological effluvium occurs when daily hair loss far exceeds this limit [3].

**Fig. 1:**
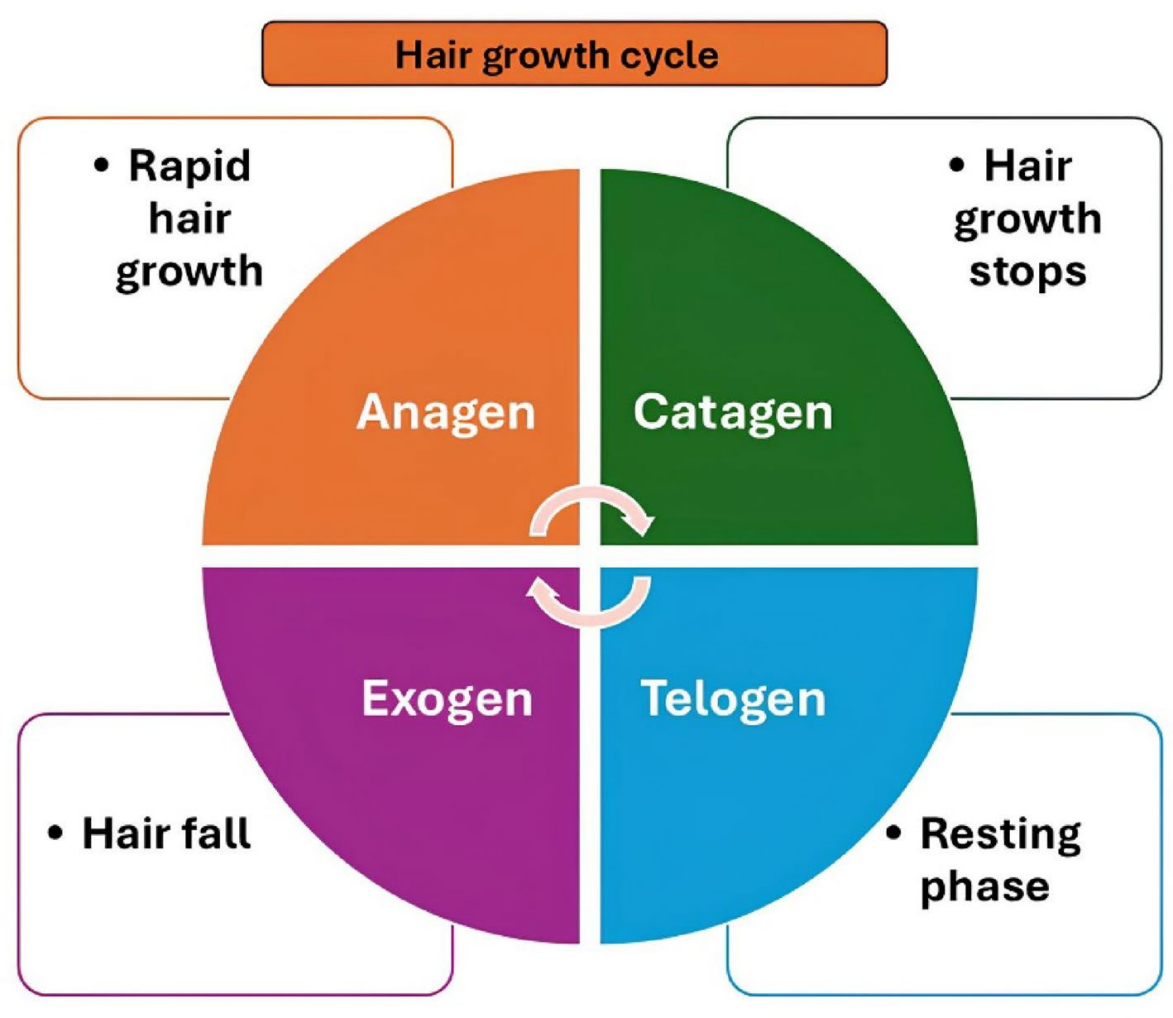
Schematic diagram presenting the hair growth cycle

The hair growth cycle consists of four phases namely, anagen, catagen, telogen, and exogen as presented in Fig. 1. Firstly, anagen begins 1 month later after birth and it is representative of hair growth. In which hair follicles are produced by stem cells proliferation, it lasts for 2–8 years [1, 2]. The next and quickest crossover stage is catagen which happens at the end of the anagen phase. Mitosis starts to slow down and eventually stops at the end of the anagen phase. Apoptosis occurs in the lower follicles. The basement membrane that surrounds the follicles thickens and forms a glassy membrane. This phase lasts a few weeks and affects 1–3% of all hairs [4]. Next, is the telogen phase or resting stage in which the hair follicles phase loses hair. Finally, it is the exogen in which the lost hair shafts can be fall [5].

Although these four stages constitute a normal cycle, alopecia throws off this rhythm, either by shortening the anagen phase or keeping the follicle from returning to it. The goal of conventional topical therapies is to address these disruptions; however, they often fail to reach the hair bulb deep inside the dermis due to limited skin penetration and quick clearance. This gap between the physiological needs of the follicle and the limitations of standard therapies has shifted the focus toward more sophisticated delivery mechanisms.

Nanocarrier-based formulations represent a promising new approach for treating alopecia. These formulations improve treatment effectiveness by increasing drug availability and therapeutic effect. They achieve this through sustained release, better skin penetration, and targeted delivery to hair follicles. Furthermore, unlike traditional treatments, nanocarrier systems offer advantages such as being painless, non- or minimally invasive, potentially reducing the need for long-term treatment, and minimizing the chance of the condition returning after stopping treatment [6]. Lipid-based nanoparticles (LNPs) represent a promising advanced drug delivery system for the treatment of various types of alopecia. Their ability to encapsulate active pharmaceutical ingredients (APIs) and deliver them specifically to the hair follicles offers potential advantages over conventional topical formulations. LNPs demonstrated an excellent bio-compatibility, they can provide sustained or controlled drug release, offer enhanced therapeutic efficacy while reduce potential side effects. Also, the possible large-scale production and sterilization of lipid-based nanoparticles is favored for pharmaceutical manufacturing. Their well-documented scalability ensures easier transition from lab-scale formulations to large-scale commercial production [7].

This review article presents full insight on alopecia, its types and treatment models along with the potential use of lipidic nanoparticles to reach a better effect. Different types of lipidic nanoparticles were described and compared. Also, the translational route from research to patents then to market is also outlined with the faced challenges. Unlike previous reviews that often focus solely on a certain section, this review uniquely integrates them.

## Types of alopecia and its etiology

**Fig. 2:**
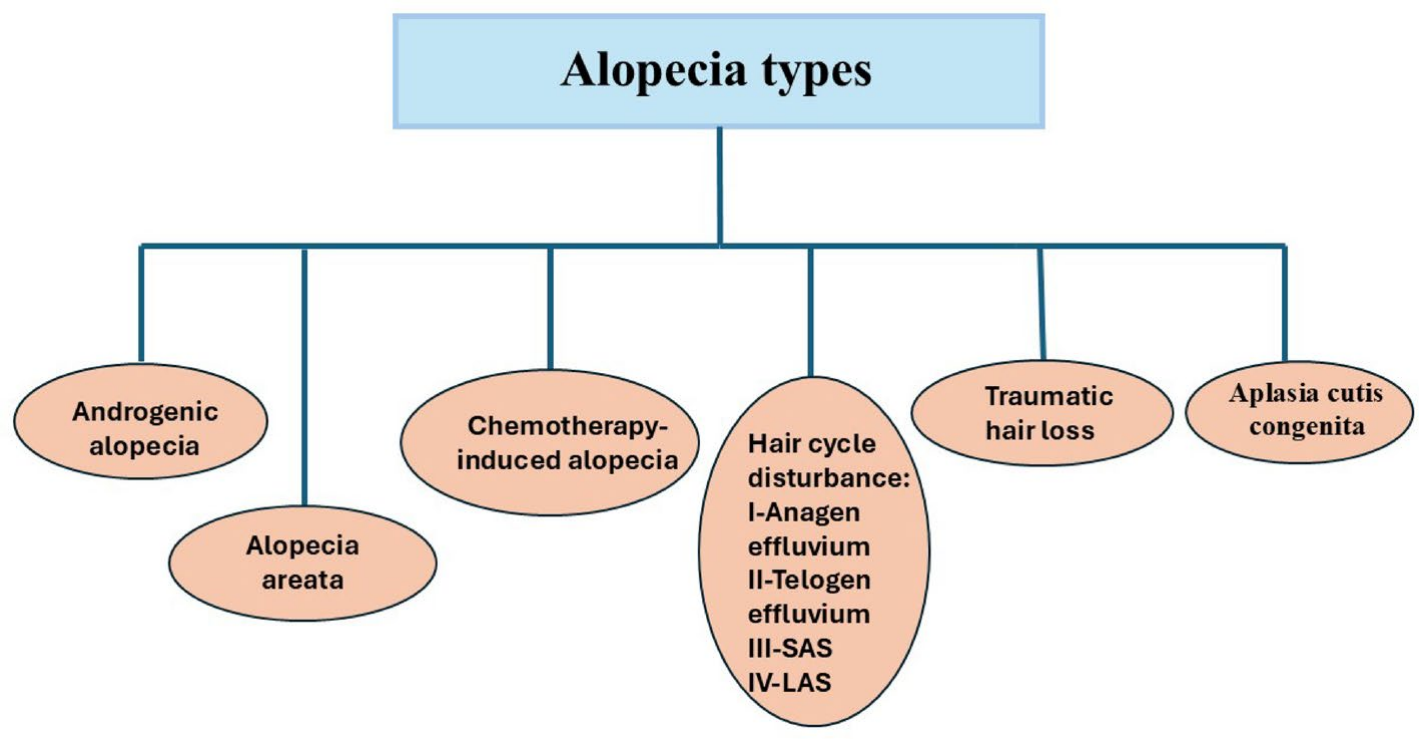
Schematic diagram presenting the different types of alopecia

The most popular types of alopecia are androgenic alopecia, alopecia areata, chemotherapy- induced alopecia (CIA), anagen effluvium, telogen effluvium, traction alopecia, and trichotillomania (Fig. 2) [2]. They can be categorized into two groups such as scarring alopecia, which is characterized by a swelling reflex to the injured follicle, and non-scarring alopecia triggered by multiple causes such as hormones, medication, nutrition, and certain illnesses [8] (Fig. 2).

### Androgenic alopecia

Androgenic alopecia is the most common type of hair loss and is associated with androgen-mediated follicular reduction. Binding of androgens to follicular receptors shortens the anagen phase and prolongs the telogen phase, leading to progressive hair thinning and follicular shrinkage [9].

### Alopecia areata (AA)

Alopecia areata is an autoimmune disorder characterized by immune-mediated attack on anagen hair follicles, resulting in localized or generalized hair loss [10]. Its pathogenesis involves dysregulation of immune pathways associated with T-lymphocyte activation, including human leukocyte antigen (HLA), natural killer cell activating ligands, interleukins, and cytotoxic T-lymphocyte-associated antigen-4 (CTLA-4). These immune reactions promote inflammatory damage to hair follicles and disrupt normal hair cycling [11].

### Chemotherapy-induced alopecia

One of the common side effects of chemotherapy is hair loss, which can negatively affect patients psychologically and may lead to depression [12]. Chemotherapeutic agents primarily target rapidly proliferating cells, including non-neoplastic cells such as hair fiber keratinocytes during the growing phase. Hair matrix keratinocytes in the anagen phase are considered among the fastest proliferating cells in the body. Consequently, exposure to chemotherapeutic agents induces apoptosis of hair follicles in the anagen phase, leading to hair breakage and loss. In addition, hair follicles shift to the telogen phase following chemotherapy exposure, accompanied by prolongation of the telogen phase and shortening of the anagen phase [13].

### Hair cycle disturbances

Hair cycle disturbances represent a group of non-scarring alopecia resulting from disruption of normal hair growth cycling. *Anagen effluvium* is characterized by abrupt cessation of mitotic activity in anagen follicles, most commonly following exposure to chemotherapeutic agents or other cytotoxic drugs, leading to rapid hair shedding during the anagen phase [14]. *Telogen effluvium* is a diffuse, non-inflammatory form of hair loss that typically follows a physiological or psychological trigger such as stress, childbirth, weight loss, or drug exposure (e.g., interferons, antihyperlipidemic agents, retinoids, and anticoagulants), resulting in premature transition of follicles into the telogen phase [15]. *Short anagen syndrome (SAS)* is a rare condition characterized by reduction in anagen duration, leading to persistently short, fine hair, and is most commonly observed in children, with occasional autosomal dominant inheritance patterns [16].

### Traumatic hair loss

Traumatic alopecia includes hair loss resulting from physical or mechanical injury to hair follicles. *Trichotillomania* is a compulsive disorder characterized by repetitive self-pulling of hair, leading to irregular patches of hair loss, most commonly seen in children and individuals with psychiatric conditions [17]. *Traction alopecia* results from prolonged or repeated tension on the hair shaft due to certain hairstyling practices such as tight braiding, ponytails, and the frequent use of chemical or styling procedures, ultimately causing hair breakage and follicular damage [18].

### Aplasia cutis congenita

It is an uncommon congenital condition defined by localized absence of skin present at birth, most frequently affecting the scalp and often resulting in permanent scarring alopecia. The extent and pattern of the skin defect differ between patients and may occur as result of congenital malformations. Associated abnormalities can include chromosomal disorders such as trisomy 13 or 4p deletion syndrome, as well as craniofacial defects including cleft lip and palate [19].

## Treatment of alopecia

There are many types of therapeutic agents for controlling alopecia but only few drugs are approved by the FDA for hair re-growth. Many treatments are reported to have side effects or inadequate results. Regarding varied alopecia types, alopecia treatment should be precisely prescribed for a certain type targeting a specific site of action.

### Approved drugs used for alopecia treatment

#### Minoxidil (Regaine®)

Minoxidil is approved by the FDA organization for topical treatment of hair fall by causing the opening to the potassium channel, which causes hyperpolarization to the cell membrane followed by vasodilation, and angiogenesis as well as potassium channels opening, all this together facilitates nutrition to the hair follicle by increasing oxygen entrance and blood supply. Moreover, it’s responsible for converting hair follicles from telogen phase to anagen phase again [20].

While major side effects, such as pericardial effusion, are uncommon at doses used for alopecia, common side effects include dose-dependent hypertrichosis (24% incidence), temporary shedding (16–22% incidence), and moderate peripheral edema (2%) [21].

#### Finasteride (Propecia®)

The second FDA approved drug for hair regrowth is Finasteride, but this one is used mainly for androgenic alopecia owing to its action. The main action is downregulating the expression of 5 alpha reductase on the androgen receptors thus inhibiting action of dihydrotestosterone (DHT). It should be administered early before the hair follicle damages. It should be avoided for females especially pregnant female due to its teratogenic action [22].

Clinical studies attest to finasteride’s effectiveness in increasing hair density and count, especially in the vertex and mid-scalp areas. Many users experience sustained hair regrowth after using the product for at least 2 years. According to some research, finasteride may work better when combined with other topical medications [23].

#### Dutasteride (Avodart®)

A dual inhibitor of 5-alpha-reductase (type 1 and type 2), dutasteride is a possible substitute for finasteride and has shown to be more effective in treating androgenetic alopecia. When compared to finasteride, it is 100 times more effective at blocking 5-alpha-reductase type 1 and three times more effective at blocking type 2. Tablets containing 0.5 mg of dutasteride are available to treat androgenetic alopecia [24].

Regarding effectiveness, research shows that AGA patients experience an apparent boost in hair regrowth and maintenance compared to finasteride. Recent studies have investigated its efficacy in treating female pattern hair loss using other therapies [23].

#### Baricitinib (Olumiant®)

It is a Janus kinase (JAK) pathway inhibitor taken as a daily pill, it was approved by the FDA in 2022 for adults with severe alopecia areata [25]. Janus kinase inhibitors (JAKi), such as baricitinib, inhibit the inflammatory Janus kinase-signal transducer and activator of transcription (JAK-STAT) signaling pathway, which is involved in cytotoxic T cell responses that target hair follicles. This prevents hair loss and encourages hair regrowth. JAKi’s introduction has revolutionized how severe AA is treated [26].

#### Ritlecitinib (Litfulo®)

Litfulo is also a JAK inhibitor taken orally once daily, it is the first approved treatment for people under the age of 18. In a clinical trial, treatment with Litfulo was significantly more effective in achieving hair regrowth reaching 80% or more scalp hair coverage compared to placebo. In 2023, the FDA approved ritlecitinib for the treatment of severe alopecia areata in adults and adolescents aged 12 and up [27].

For AA patients to experience the best results, long-term treatment may be necessary, especially if they have significant hair loss. According to the analysis, many patients must receive ritlecitinib therapy for more than 6 months to achieve the desired clinical response, even though some patients may experience clinically significant hair regrowth at 6 months [28].

#### Deuruxolitinib (Leqselvi®)

It is also a JAK inhibitor which received FDA approval in 2024 to treat alopecia areata in adults. It is taken orally twice daily [29]. Serious side effects were rare in the deuruxolitinib group, but headache, acne, and elevated blood creatine phosphokinase were experienced by ≥ 5% of patients [30].

Beside those drugs, there are some other trends for the treatment of alopecia such as herbal therapies, surgical intervention, and nutrition.

### Herbal therapies for treatment of alopecia

There are many herbal medications that are not licensed for the treatment of alopecia. Various research has demonstrated that several phytochemicals have the capacity to stimulate hair growth in in-vitro and in-vivo models worth [1, 2]. Herbal remedies are used topically or systemically to prevent hair loss while having lower side effects than synthetic treatments. For example, Cucurbita pepo (pumpkin), Curcuma aeruginosa (pink and blue ginger), Trifolium pratense (red clover), Serenoa repens (palmetto), and Panax ginseng (Chinese red ginseng) are the plants with the most proven effects on alopecia. The assumed mechanism of action is primarily due to the inhibition of 5α-reductase, in addition to nutritional support and scalp blood circulation enhancement [31].

### Nutrition and supplements

Nutrition has a main role in hair growth and to be healthy. Many nutrients are required for hairs to grow in a healthy status such as zinc, iron, selenium, calcium, potassium, magnesium and copper in addition to vitamin A, B, C, D, and E. All these nutrients play an essential role in hair growth by increasing blood supply to hair follicles, preventing free radicals and oxidation, and facilitating follicle cycle [32].

### Potential future treatment for alopecia

Discovering a novel medication for alopecia together with the use of advanced technology of drug delivery can lead to a better prevention of hair loss. Future alopecia treatments may include the identification of active hypertrichotic phytochemicals, integrated medications, gene therapy, and stem cell therapy [33]. On the other hand, under the strategy of drug repurposing, some drugs have proven efficacy in reducing hair loss, below are some examples:

#### Latanoprost

The increased eyelash and eyebrow hair growth in glaucoma patients led to the use of prostaglandin analogs like latanoprost for the treatment alopecia. Hair growth is induced and the anagen phase is prolonged by prostaglandin F2 (PGF2) and PGE2, while PGD2 inhibits hair growth [24].

Ocular hypertension and glaucoma are frequently treated with latanoprost (LAT), an analog of prostaglandin F2 alpha (PGF2α). Most of its negative effects, such hypertrichosis of the lashes, are categorized as minor. Prostaglandin signaling may be involved in hair growth, according to promising findings from alopecia clinical trials and animal models. The biological mechanism by which it promotes hair growth, however, is poorly understood [34].

#### Spironolactone

Spironolactone is an antiandrogen and diuretic that spares potassium. It inhibits 17, 20 lyase, and 17-alpha hydroxylase, which reduces testosterone production. It is an excellent treatment for female pattern hair loss (FPHL) due to its antiandrogen qualities, but it is not recommended for androgenetic alopecia (AGA). FPHL is typically taken at a dose of 12.5–200 mg per day growth [24]. Hyperkalemia, irregular menstruation, breast tenderness, exhaustion, and lightheadedness are some of its side effects and possible negative consequences. These problems might be lessened by more recent formulations [35].

Because of its feminizing effects, it is inappropriate for men. Sustained benefits require long-term use. Alternative anti-androgen treatments with fewer hormonal side effects are still being researched [36].

#### Bicalutamide and flutamide

Flutamide is an effective antiandrogen that blocks androgen receptors, a dose of 250 mg per day has demonstrated to be effective in hyperandrogenic women with female pattern hair loss. According to a case report, a patient with female pattern hair loss unresponsive to topical minoxidil and spironolactone showed a reversed hair loss by flutamide [24]. In the same context, bicalutamide can be used for the treatment of hair loss.

Even though low dosages of antiandrogen medications are usually used to treat hair loss, side effects from peripheral androgen inhibition/downregulation or hyperestrogenism are still possible. Among them are depression, gynecomastia, menstrual irregularities, decreased libido, and sexual dysfunction [37].

#### Corticosteroids

For alopecia areata (AA), a variety of topical corticosteroid formulations, such as creams, gels, ointments, lotions, and foams, have been used and showed different efficacy. Compared to 27% in the group that received 0.05% betamethasone dipropionate lotion, 61% of patients who used 0.1% betamethasone valerate foam experienced more than 75% hair regrowth [11].

Skin atrophy and systemic effects, including weight gain, high blood pressure, and osteoporosis, are among the negative impacts. Advanced delivery techniques aim to mitigate these risks [38].

Follicular drug penetration is mediated by a complex interplay of anatomical characteristics (density, morphometry, and functional status), the physicochemical properties of both the permeant and its vehicle, and external application variables (pre-treatment, mechanical manipulation, and environmental conditions [39].

To develop effective therapeutic systems for alopecia, it’s crucial to overcome the challenges posed by the body’s natural defenses. This involves the normal structure of hair follicles (anatomical barriers), the blood flow to the scalp changes in healthy and diseased states (physiological barriers), and the alterations occurring at a cellular and molecular level in affected tissues (pathophysiological barriers). These obstacles can be defeated by the development of nanotechnology-based delivery system including lipidic nanoformulations.

Given the limitations associated with conventional alopecia therapies, considerable attention has recently been directed toward advanced drug delivery systems capable of enhancing therapeutic efficacy and follicular targeting. Among these approaches, lipid-based nanocarriers have emerged as promising platforms for improving drug penetration, retention, and localized delivery to hair follicles.

## Application of lipid based nanoparticles in alopecia

Lipid nanoparticles represent a versatile and effective class of delivery systems for therapeutic agents, particularly in the treatment of alopecia [40–42]. Their inherent biocompatibility, capacity to encapsulate a range of drug molecules, and ability to facilitate controlled and targeted release make them ideal candidates for enhancing hair regrowth treatments [43]. The different types of lipid nanoparticles; such as solid lipid nanoparticles (SLNs), nanostructured lipid carriers (NLCs), liposomes, transferosomes, and nanoemulsions, offer distinct advantages and mechanisms of action. These properties can be harnessed to improve the delivery and efficacy of treatments for hair loss. This section will provide an overview of some types of lipid nanoparticles, examining their composition, functional benefits, and specific applications in the context of alopecia therapy.

### Solid lipid nanoparticles (SLNs)

SLNs are a key category of lipid based drug delivery systems, recognized for their utility in various medical applications, including alopecia treatment [44]. These solid core lipid nanoparticles are composed of solid lipids ranging from 0.1% to 30% w/w distributed in a water phase, which includes a surfactant (0.5–5% w/w) [44]. They are able to encapsulate both hydrophobic and hydrophilic moieties [45]. They are one of the best options for medicine delivery since they can be composed of biocompatible components allowing targeting ability [45]. SLNs offer several advantages when applied topically, such as safety, tolerability, sustained release profile, increased skin penetration, less systemic absorption with associated side effects [44]. The small particle size of SLNs enhances their penetration and distribution in the scalp, optimizing the delivery of therapeutic agents directly to the sites of hair loss [41].

Many relevant studies have demonstrated the therapeutic potential of drugs loaded SLNs in the treatment of alopecia.

A study on Roxithromycin loaded lipid nanoparticles investigates the targeting activity of SLNs to improve drug delivery to hair follicles [46]. This study aimed to effectively integrate ROX into SLN for the purpose of assessing its efficacy as a follicular targeting system with the potential to treat androgenetic alopecia. The selected formulation of ROX loaded SLNs prepared using glyceryl behenate as the solid lipid, exhibited a mean particle size of 172 ± 2 nm with a PDI value of 0.237 ± 0.007, indicating a uniform particle distribution suitable for follicular targeting. The ZP was measured at − 31.7 ± 3.1 mV, reflecting particles stability. The formulation achieved a high entrapment efficiency of 82.1 ± 3.0% ensuring efficient drug loading within the lipid matrix. These physicochemical properties collectively proved the possibility of penetration to human pilosebaceous units and delivering ROX, as confirmed by ex-vivo penetration studies and cyanoacrylate follicular biopsy. The formulation also demonstrated long term physical stability over 26 weeks possessing no evidence of skin irritation, ensuring its potential as a safe and effective system for topical follicular delivery.

Hamishehkar et al., developed SLNs loaded with the anti-androgen agent (Flutamide) and evaluated their follicular targeting [47]. Results revealed that after 2 months’ storage, the optimized Flutamide-loaded SLNs (size ≈ 198 nm, encapsulation efficiency percentage ≈ 65%, and loading efficiency percentage ≈ 3.27%) demonstrated good stability. Flutamide was shown to be in an amorphous state by X-ray diffraction studies, indicating homogeneous drug dispersion throughout the structure of the SLNs. A better localization of the medication in the skin was demonstrated by the SLN formulation’s which had a higher skin drug deposition (1.75 times) compared to Flutamide hydroalcoholic solution. Also, the in-vivo hamster model study demonstrated a greater growth of new hair follicles than flutamide hydroalcoholic solution, this could be because the SLNs accumulate more in the hair follicles and because the drug is released gradually and continuously through the SLNs thereby increasing the exposure time of the hair follicles to the drug.

### Nanostructured lipid carriers (NLCs)

NLCs are an advanced form of lipid nanoparticles developed to address some of the limitations seen in SLNs [48]. They are composed of a blend of solid and liquid lipids, which enhances their ability to encapsulate a variety of therapeutic agents more effectively by allowing additional space for drug loading and improves the stability of the formulation [44]. Moreover, the presence of liquid lipids may contribute to improved flexibility of the lipid matrix, which can enhance drug release behavior and facilitate better interaction with the stratum corneum, potentially improving skin and follicular penetration. This unique composition allows for higher drug loading and better controlled release properties, making NLCs particularly suitable for application in alopecia treatment. Indeed, scientists have exploited these features in preparing different NLCs formulations for the treatment of alopecia [41, 49, 50].

Minoxidil-encapsulated NLCs were made by Wang et al. using stearic acid as the solid lipid and oleic acid as the liquid lipid. The optimized minoxidil loaded NLCs exhibited a mean PS of 281.4 nm, a PDI value of 0.2 and − 32.9 mV for the ZP. The drug showed high entrapment efficiency and loading capacity of 92.5% and 13.8%, respectively. Unlike the SLNs, minoxidil-NLCs demonstrated a superior storage stability and 10.7-fold increased drug retention in the skin. Because of their ability to fuse with sebum or awaken dormant follicles, NLCs have been shown to broadly enhance drug deposition in follicles and considered to be a promising vehicle for topical minoxidil delivery [51].

Another study was done by Aljuffali et al., to demonstrate the efficiency of modified NLCs loaded minoxidil named squarticles. Squarticles are composed sebum mimetic lipids such as squalene and hexadecyl palmitate with a targeting moiety of anti-PDGF receptor β antibody to target the dermal papilla cells (DPCs) and follicles [52]. Authors studied the difference in physicochemical properties of plain squarticles versus squarticles conjugated to anti-PDFG-β receptor antibodies, demonstrating that the antibody targeted formulation significantly enhanced follicular delivery and cellular uptake by DPCs. Results revealed that the mean diameter of the antibody-free squarticles was 236 nm. The particle size decreased to 195 nm when PDGF antibody was added. A limited size distribution was suggested by the polydispersity (PDI) values of 0.29 and 0.19 for squarticles and PDGF-squarticles, respectively. The MXD loading capacity of both nanocarriers was around 50%. Regarding the in-vivo skin penetration study in mice, encapsulated minoxidil was deposited in the follicles and entered the DPCs, where it increased the expression of proteins linked to angiogenesis and sped up cell proliferation. The formulated NLCs were shown to triple the concentration of minoxidil in follicles when compared to the solution group.

In another study, nanostructured lipid carriers (NLC) was used to deliver minoxidil and latanoprost directly to hair follicles. Chemical compatibility of the drug combination was confirmed using thermal analysis and FTIR. The resulting NLC, with a size of approximately 393 nm and a positive charge, efficiently encapsulated 86.9% of minoxidil and 99.9% of latanoprost. In-vitro studies on human keratinocytes demonstrated that the drug combination, especially when delivered via NLC, promoted cell growth, migration, and increased expression of key hair growth markers (MKI67 and VEGF). The NLC formulation was well-tolerated in safety tests (reconstructed human epidermis), demonstrating excellent skin tolerance with no reported irritation. Penetration studies on porcine skin showed that the NLC effectively targeted hair follicles, significantly increasing the delivery of both drugs to these structures compared to the free drug combination. This targeted delivery achieved approximately 50% penetration of both drugs into the follicles. These findings suggest that NLC-mediated delivery of minoxidil and latanoprost offers a promising approach for alopecia treatment [41].

Shamma et al., enhanced the effectiveness and safety of antihypertensive drug spironolactone in the treatment of alopecia by creating colloidal nanostructured lipid carriers (NLCs) that facilitate follicular drug transport [53]. According to author’s findings, the optimized formulation formed of compritol 888 and 5% liquid lipid (1:1, olive oil and transcutol) exhibited an EE of 87.36%, PS of 215.6 nm, and − 18.7 mV for the ZP. Confocal laser scanning microscopy confirmed that the prepared formulation exhibited high penetration and accumulation in the hair follicles of albino mice. Results revealed a broad distribution of fluorescence surrounding the hair shafts and follicles, most likely as a result of the NLCs accumulation in this region. This indicates that the hair follicles may serve as a potential pathway for NLC penetration. Additionally, there was fluorescence deposition at the hair structure’s outermost layer, most likely as a result of the NLCs and hair cuticle interacting. These results suggest that spironolactone loaded NLCs offer a promising localized therapy for androgenic alopecia by increasing follicular drug delivery while minimizing systemic exposure.

Recently, Qibin et al., created topical formulations of roughly 200 nm-sized tofacitinib-loaded cationic lipid nanoparticles (TFB-cNLPs) and a positive zeta potential, which facilitated strong interaction with the negatively charged skin surface, promoting percutaneous absorption. The lipid matrix consisted of a mixture of cationic lipids, stearic acid, Gelucire 50/13, or medium-chain triglycerides (MCT), which together provided structural stability, efficient drug loading, and enhanced skin permeation. The ex-vivo study on pig ear skin demonstrated that TFB-cNLCs improved transfollicle targeting and transepidermal permeability. Due to presence of liquid lipids, nanoparticles become more lipophilic, which facilitates their entry into hair follicles. Positively charged surface modification improves transport via the transfollicular and transepidermal pathways, increasing delivery efficacy. Because of their greater density, nanoparticles have a propensity to enter the channels deeper when they are in contact with sebum. Additionally, the in-vitro follicle model performed by the authors showed that TFB-cNLP prevented the Janus kinase/signal transducers and activators of transcription (JAK/STAT) pathway, thereby reducing the symptoms of alopecia areata caused by interferon gamma (IFN-γ). Furthermore, in the C3H mouse model of alopecia areata, it also decreased the number of cytotoxic CD8 + NKG2D + T cells in-vivo, which prevented the disease’s progression and reversed hair loss. According to these results, TFB-cNLP improved hair follicle targeting supporting its potential as a localized, non-invasive therapeutic approach for alopecia areata [54] (Some differences between SLNs and NLCs are displayed in Table 1).

**Table 1:** Comparative table clarifying some differences between SLNs and NLCs in literature focused on alopecia treatment

Type of carrier	Loaded drug	Encapsulation efficiency (EE)	Penetration depth and targeting outcome	References
SLN	Roxithromycin (ROX)	82.1%	Successful penetration of human pilosebaceous units; confirmed follicular targeting	[36]
SLN	Flutamide	65%	1.75 higher skin drug deposition vs. solution; accumulation in hair follicles	[37]
NLC	Minoxidil	92.50%	10.7-fold increase in skin retention; broad enhancement of follicular deposition	[49]
NLC	Minoxidil (Squarticles)	50%	Targeted Dermal Papilla Cells (DPCs); tripled drug concentration in follicles	[50]
NLC	Minoxidil & Latanoprost	86.9% (MXD)/99.9% (LAT)	Effectively targeted follicles with ≈50% penetration into the structure	[39]
NLC	Spironolactone	87.36%	High accumulation in follicles; fluorescence observed surrounding hair shafts	[51]
NLC	Tofacitinib (cationic)	Efficient	Improved transfollicle targeting; deep channel entry via interaction with sebum	[52]

On another hand, SLN and NLC were also used to improve the effect of some herbal or plant-derived active agents against alopecia, some are listed in the following table (Table 2).

**Table 2:** Some examples of SLN and NLC used as carrier for herbal actives stimulating hair growth

Type of carrier	Composition of the carrier	Active agent(s)	References
NLC	Soya lecithin and Tween 80	*Trigonella foenum greacum* (Fenugreek) seed extract	[55]
NLC	Glyceryl mono stearate and virgin coconut oil	β-sitosterol (Plant-derived 5 α-reductase inhibitor)	[56]
NLC	Stearic acid, oleic acid, polysorbate 80 and Glycerin	Cinchonine	[57]
SLN	Precirol and Poloxamer	*Platycladus orientalis* extract	[58]

### Liposomal carriers

Many types of liposomal carriers have found successful application in the treatment of alopecia, some of them are described below.

#### Liposomes

Liposomes are spherical vesicles composed of one or more phospholipid bilayers, which can encapsulate both hydrophilic and hydrophobic active agents [59]. Their unique structure allows for the efficient delivery for therapeutics, improving their stability and bioavailability [60]. In the context of alopecia treatment, liposomes are particularly beneficial due to their ability to target hair follicles directly [61], enhancing the delivery and effectiveness of active ingredients. The biocompatibility and non-toxic nature of liposomes make them ideal for topical application [62], reducing the risk of side effects. Additionally, their lipid composition is similar to that of the epidermis, which allows them to thoroughly permeate the epidermal barrier. Most liposomes that are applied topically to the skin accumulate in the stratum corneum’s upper layers, acting more as a “reservoir” to provide a more localized effect [63, 64].

Many studies were performed to evaluate the effect of liposomal nanocarriers and their use in the field of alopecia management and hair growth promotion.

A study performed by Xu etal., to create the unique FGF-2-LIP-SF hydrogel [65]. They encapsulated the fibroblast growth factor (FGF-2) into liposomes (FGF-2-LIP) followed by its incorporation into silk fibroin (SF). They found that when the drug/lipid ratio was greater than 1:300, the encapsulating effectiveness of FGF-2 was over 90%. The results demonstrated that the encapsulated FGF-2 was able to get into the dermis after application of FGF-2-LIP-SF on the mice skin using the confocal laser scanning microscopy (CLSM). The hair of mice treated with testosterone (TES) grew back quickly after receiving treatment with FGF-2-LIP-SF hydrogel, and the hair follicles also reached the anagen phase. Furthermore, the application of FGF-2-LIP-SF hydrogel resulted in a significant inhibition of the expression of cytokines linked to inflammation, including TNF-α and IL-6. Meanwhile, FGF-2-LIP-SF hydrogel stimulated the production of CD133, a marker of epidermal stem cells, in hair follicles. This study stated that FGF-2-LIP-SF may be a viable treatment choice for alopecia areata.

Cholesterol, the major steroid usually used in liposomes preparation, is a precursor for the manufacture of testosterone [66], which can then turn into dihydrotestosterone that can damage hair follicles and exacerbate hair loss [67]. In order to overcome this drawback, Zhang et al., created a cholesterol-free liposome (PPD-Lip) by substituting cholesterol with protopanaxadiol (PPD) [68]. Results showed that PPD-Lip encouraged the proliferation and migration of dermal papilla cells, upregulated the mRNA levels of positive regulators linked to hair growth, and sped up angiogenesis in-vitro. While, in -vivo, it stimulated hair growth in models of androgenetic and telogen alopecia in mice. Furthermore, this study demonstrated that PPD-Lip loaded with dutasteride, demonstrated a greater efficacy in treating androgenetic alopecia.

Newly, a novel transdermal delivery system formed of hyaluronic acid liposome loaded with minoxidil and nitric oxide (HL@Mi/NONOate) was constructed for the treatment of androgenetic alopecia [69]. The lipid vesicles were composed of soybean lecithin coated with hyaluronic acid to enhance skin adhesion and prolong residence time, while the NO was incorporated in the form of chemically modified donor (NONOate), enabling sustained, and controlled release. The vesicles exhibited a particle size of approximately 200 nm (based on TEM), and a moderate negative zeta potential, contributing to colloidal stability and efficient skin penetration. The authors succeeded to formulate a promising transdermal and hair-regrowth effects for both nitric oxide and minoxidil. Comprehensive mechanistic investigations have identified three possible mechanisms for the synergistic androgenetic alopecia therapy. Initially, nitric oxide caused capillaries dilatation and accelerated blood flow, which allowed minoxidil to enter the body efficiently. Additionally, HL@Mi/NONOate increased the expression of regulatory factors related to follicular stem cells and encouraged angiogenesis and cell proliferation.

#### Transferosomes

Transferosomes are another type of liposomal carriers investigated in literature for the management of alopecia. They are a specialized type of liposome known for their highly deformable and flexible structure [70]. In order to minimize membrane stiffness and increase their deformability, the transfersomes embed edge activators into lipid bilayer membranes. This prevents vesicle rupture during drug administration and enables vesicles to transport medications from the epidermis to deeper regions [71, 72].This distinctive characteristic lets them to penetrate deeper into the skin by squeezing through narrow pores, enhancing their efficiency [73]. In alopecia treatment, they can significantly improve the delivery of active moieties to the hair follicles, and consequently enhance treatment efficacy. Their ability to cross the skin’s barrier more efficiently than conventional liposomes makes them a valuable tool in promoting hair regrowth [74].

Owing to the adverse effects of finasteride when taken orally, Ahmed et al. proposed using nano-transferosomal gel to treat androgenetic alopecia; in order to improve the drug’s accumulation in hair follicles as well as overcoming its oral side effects [75]. The transferosomes were composed of phosphatidylcholine and a suitable edge activator, enabling enhanced flexibility and skin penetration. The optimized formulation showed a particle size of approximately 130 nm with a low PDI value, indicating a uniform vesicle population, and ZP around − 32 mV, suggesting good stability. Finasteride loaded transferosomes were incorporated into carbopol 940 gel for topical application. Ex-vivo skin permeation and follicular targeting study revealed consistent fluorescence (rhodamine) intensity across the rat skin for the nanoformula compared with the control gel formula, indicating enhanced transport through skin layers.

Another study done by Sun et al., aimed to create a transfersomal formulation of minoxidil and tocopherol acetate as an approach to enhance follicular targeting and therapeutic efficacy in the management of androgenetic alopecia [76]. The formulation aimed to exploit the synergistic effect of minoxidil’s vasodilatory effect and tocopherol acetate’s antioxidant activity in order to reshape the hair follicle microenvironment. The transferosomal physicochemical properties, such as PS, ZP, and lyophilicity, are optimized to improve skin permeation and facilitate drug accumulation within the hair follicles. Results showed that transferosomes gel demonstrated superior in vitro transdermal penetration ability and hair growth-promoting capacity in C57BL/6 mice compared to commercial formulations. In addition to being non-irritating, skin adherent, stable, with a high ability to promote hair growth, making it a potentially effective topical therapeutic option for alopecia.

Lately, Liu et al., explore a novel treatment for androgenic alopecia [77]. The study investigates the combined effect of ginsenoside Rg3 and minoxidil, delivered via transferosomes on hair growth in C57BL/6 mice, these showed a significant improvement in hair regeneration compared to treatments with minoxidil alone. Authors suggest that the addition of ginsenoside Rg3 enhances minoxidil therapeutic effect by multiple mechanisms such as preventing testosterone from being converted to dihydrotestosterone and lower the amount of inflammatory markers. Thus enabling Rg3 to have a synergistic effect on minoxidil treatment for androgenic alopecia. Other types of liposomal carriers were described in researches focused on alopecia, some are listed in the table below (Table 3).

**Table 3:** Type of liposomal carriers for alopecia treatment

Liposomal carrier type	Composition	Key benefits	Applications in alopecia	References
Niosomes (NIS)	Finasteride (FIN), polyoxyethylene stearate, span 60 and ethanol	Overcome the restrictions associated with orally administered finasteride	In vitro skin permeation study revealed that the total drug retention of FIN-NIS in the full skin layer (52.61 ± 0.01 μg/cm^2^) was 4.1 and 2 times higher than that of FIN-suspension (12.95 ± 0.09 μg/cm^2^) and FIN-hydroalcohol (26.9 ± 0.02 μg/cm^2^), respectively. This Improvement of the drug’s retention in hair follicles of androgenic mice resulting in promoting hair regeneration, and decreasing side effects	[78]
Proniosomes	Finasteride, cholesterol, span 60, and ethanol	Enhancement of transfollicular delivery of finasteride	Increase of number and size of hair follicles in mice model through inhibition of type I 5α-reductase, which could help control hair loss without having any negative side effects compared to the control group	[79]
Glycerosomes	Minoxidil, phospholipid, cholesterol, and glycerol	Enhancement of skin penetration and therapeutic effect of minoxidil in treating hair loss	Presence of glycerol increase flexibility and fluidity of liposomal bilayer leading to improved skin penetration and localization of minoxidil around the hair follicle resulting in promoting the growth phase of the hair follicle	[80]
Transethosomes	Minoxidil, phospholipid, oleic acid, and ethanol	Maximization minoxidil efficacy in the management of androgenic alopecia as well as decreasing its side effects	The presence of phospholipids and ethanol in the vesicles interact with the lipids in the stratum corneum, changing the intercellular lipid lamellae and increasing drug permeability. Additionally, oleic acid improved stratum corneum fluidity, drug permeability, and epidermis/dermis deposition of minoxidil	[81]

Liposomal nanocarriers have gained success in industrial application. Some liposomal market products for the treatment of alopecia are displayed below in Table 4 [82].

**Table 4:** Some liposomal market products

Product name	Active constituent	Company
Seskavel mulberry anti-hair loss foam	Mulberry extract, L-carnitine, panthenol	Sesderma
Identik floral repair	*Punica granatum* seed extract, lavender oil extracts	Identik Paris
Lipogaine (shampoo)	Biotin, herbal blend	Lipogaine
Revita high-performance hair stimulating shampoo and conditioner	Procyanidin B2, Caffeine	DS Laboratories
Nanominox and Nanominox-MS	Minoxidil (4%), B12, sophora flavescens extract	Sinere

### Nanoemulsions

Nanoemulsions are finely dispersed O/W or W/O emulsions with droplet sizes typically ranging from 20 to 200 nm [83]. These systems are known for their exceptional stability, optical transparency, and enhanced bioavailability of encapsulated agents [84]. They are characterized by improving the delivery of active ingredients to the scalp and hair follicles [41]. Their small droplet size allows for better penetration and absorption, making them highly effective for topical applications. Additionally, nanoemulsions can incorporate a wide range of both hydrophilic and hydrophobic substances, providing flexibility in formulation [85]. Numerous investigations were conducted to assess the effectiveness of nanoemulsions and their potential use in the treatment of alopecia and stimulation of hair growth.

A study done by Cardoso et al., demonstrated that nanoemulsions can be an effective and targeted method for delivering minoxidil and clove oil directly to hair follicles, and potentially improving the treatment of hair loss and scalp conditions [86]. Authors created a stable nanoemulsion, incorporating minoxidil and clove oil, taking advantage of the benefits of minoxidil for treating hair loss and the anti-inflammatory and antimicrobial properties of clove oil. Results demonstrated that the optimized nanoemulsions exhibited a small droplet size of 10 nm with PDI value close to 0.1, and a controlled drug release two times more efficiently than the minoxidil ethanolic solution. In addition, minoxidil skin penetration from the nanoemulsion was nine times greater than that of the traditional ethanol-based solution. The suggested clove oil-based nanoemulsions provided a promising method for the topical treatment of alopecia, as demonstrated by follicular drug penetration studies that revealed that nanoemulsions containing minoxidil entered into the hair follicles more than 26 times more efficiently than minoxidil ethanolic solution.

As known, dutasteride is a medication recognized for inhibiting 5-alpha reductase enzymes, which are implicated in hair loss by converting testosterone to dihydrotestosterone [87]. In order to improve the ability of hair follicles to store and mitigate the side effects of dutasteride, a prolonged-release nanoemulsion was prepared and, skin penetration testing revealed that it could penetrate the skin and release the drug for 6 days offering a delayed and sustained-release drug delivery [88]. The nanoemulsion did not exhibit a burst release during the first day, as only 34% of dutaseride was released within 24 h and the remaining 76% of was released in 6 days.

Another study explores the development and evaluation of a topical gel formulation combining the effect of the immunosuppressive agent cyclosporine A (CsA) and the potent antioxidant (tempol) to treat alopecia resulting from inflammation and apoptosis of hair follicles [89]. The CsA-Tempol gel formulation successfully carried CsA into the skin’s inner target layer, the dermis, according to the in-vitro permeation investigation conducted on human skin. The effect of CsA-Tempol gel on hair regeneration were further shown in the in-vivo androgenetic model performed in female C57BL/6 mice. The positive result was statistically verified through quantitative analysis of color density-measured hair regrowth and the histology analysis provided additional support for the findings.

In a very recent study, Maged et al., developed sildenafil-loaded chitosan nanocomplexes and incorporated them into a thermosensitive emulgel for topical hair loss treatment. The researchers created and characterized nanocomplexes using varying ratios of chitosan HCl and carboxymethyl chitosan. The most effective formulation (C5), made with a 1:1 ratio of the two compounds, had a particle size of 218.9 nm, a positive zeta potential, and a high drug loading of 65.8%. The chosen nanocomplex was then formulated into an emulgel using poloxamer 407 and 188 and 10% rosemary oil. This emulgel was designed to be a liquid at room temperature but to gellifie at scalp temperature. Rosemary oil was included for its known benefits and was confirmed to contain camphor, 1,8-cineole, and borneol as its major components. In hair growth study on C57BL/6 mice, the final formulation showed comparable efficacy to a market-leading minoxidil product, resulting in superior hair regeneration. The study also demonstrated that the formulation successfully upregulated key proteins (p-PI3K and VEGF) and downregulated a protein that inhibits hair growth (TGF-β), supporting its therapeutic action. This research introduces a promising, innovative, and non-invasive alternative for alopecia therapy [90].

Summarizing, nanotechnology-based lipidic drug delivery systems offer a sophisticated approach to alopecia management by leveraging diverse nanocarrier architectures to optimize follicular targeting and drug stability. Solid lipid nanoparticles (SLNs) and nanostructured lipid carriers (NLCs) excel in providing occlusive effects and high follicular accumulation, with NLCs offering superior drug loading and stability due to their less-ordered lipid matrix. While traditional liposomes provide biocompatible encapsulation, ultra-deformable transferosomes are more effective at reaching the deep follicular bulb by navigating narrow skin pores. Meanwhile, nanoemulsions serve as powerful tools for increasing the solubility of hydrophobic compounds, reducing the need for irritating solvents. By exploiting the hair follicle as a natural shunt, these systems facilitate a reservoir effect that ensures sustained release and localized action, ultimately enhancing therapeutic efficacy while reducing off-target systemic exposure [91].

## Advantages of lipid nanoparticles in alopecia treatment

As known, the existing oral treatments for alopecia are not able to adequately deliver drugs directly to the hair follicles, resulting in the occurrence of systemic side effects [92], and a lack of usefulness [93]. Nonetheless, the tolerance of topical medicines may be impacted by common adverse effects such skin irritation and discomfort [94]. Accordingly, it is mandatory to generate an effective and nontoxic follicular drug delivery system for the topical treatment of alopecia. Recent approaches for follicular drug delivery mostly focus on using nanoformulations drug delivery systems [41]. Topically applied nano-formulations primarily target the skin’s layers, improving diffusion, retention at the intended site, and reducing systemic absorption [95, 96], this helps to prevent any negative systemic effects.

Lipid-based nanoparticles (LNs) are among the most widely used nanoformulations for alopecia treatment due to their efficacy and versatility. For dermal application of pharmaceutical and cosmetic products, LNs have several benefits, including: controlled drug release and targeting; enhanced drug physical stability; ability to incorporate hydrophilic and lipophilic drugs; and lack of need for organic solvents [42]. Their small size and lipid composition allow efficient penetration into the stratum corneum and preferential accumulation within hair follicles, enabling targeted delivery of therapeutic agents to the site of action.

Moreover, LNs have occlusive properties that facilitate medication penetration into the skin and raise the water content of the skin [97]; also, they can easily interact with the lipids of the skin [98]. Additionally, LNs shield the loaded compounds from deterioration or chemical degradation [99]. As well, surface properties of these carriers can be adjusted to improve adhesion to the skin and prolong residence time, further increasing local drug bioavailability [48].

In this section, some of the specific advantages that make lipid-based nanoparticles a superior choice for treating alopecia are discussed. Overall, the advantages of lipid-based nanoparticles in alopecia treatment are interrelated and collectively contribute to their therapeutic effectiveness, where improvements in drug stability, controlled release, follicular targeting, and biocompatibility work in synergy to enhance localized scalp delivery while minimizing systemic exposure. However, the relative contribution of each advantage may vary depending on the specific nanocarrier type and formulation design.

### Improved drug stability and protection

LNs create a stable environment for drugs, protecting them from environmental factors such as light, oxygen, and moisture [100]. This protection ensures that the active ingredients remain effective until they reach the target site, which is particularly beneficial for drugs that are prone to degradation such as minoxidil and corticosteroids.

### Controlled and sustained drug release

Lipid based nanoparticles are designed to provide controlled and sustained drug release due to their structural characteristics and composition. The solid or semi solid lipid matrix forming these nanoparticles acts as a reservoir, gradually releasing the encapsulated drug over time and reducing frequency of application [101]. This release is often diffusion controlled, allowing the drug to slowly diffuse out of the lipid matrix [99] and ensures prolonged therapeutic effects [102]. Additionally, the lipid matrix can erode slowly, further extending the release period.

### Minimized systemic side effects

By enabling localized delivery of drugs to the scalp, LNs reduce systemic exposure and decrease the risk of side effects that are common with oral medications [103]. This targeted delivery ensures that the therapeutic agent is concentrated where it is most needed, which minimizes the amount of the drug that passes into the bloodstream and affects other parts of the body. As a result, the side effects accompanying the other routes of administrations are significantly reduced, making the treatment safer and more focused on addressing hair loss effectively.

### Enhanced penetration and retention

Regarding the small size and lipid composition of nanoparticles, they can interact effectively with the lipid rich environment of the scalp and hair follicles [104]. The process of combining stratum corneum lipids of the scalp with lipid nanoparticles facilitates drug penetration into deeper tissues. Drug dispersion can be achieved uniformly by lipid nano-carriers by forming nearby contact with corneocyte island furrows and superficial connections of stratum corneum. Moreover, they provide an expansion of inter-corneocyte gaps [105]. Owing to the physiological lipid composition of the skin, SLN/NLC provide a smooth attachment to the SC during lipid rearrangement, enabling embedded drug molecules to enter the deeper layers of the skin. They have the potential of adhesiveness and occlusive capabilities that enable them to manufacture a homogeneous and uniform layer on the stratum corneum, extend residence time, and enhance skin penetration by interacting with skin layers and changing its barrier properties [106].

Additionally, their nanoscale diameter plays a role in improving the influx through the skin. However, choosing the right lipid content in relation to the physicochemical characteristics of the molecule also has a big impact on skin penetration [107]. Moreover, hair follicles especially those in the scalp, calf, and forehead regions are superior drug stores to the stratum corneum. It was found that the medications can be delivered via nanoparticles into deeper functional structures, where they can even build up for a few days [108].

### Versatility in drug encapsulation

LNs exhibit significant versatility in drug encapsulation, allowing them to effectively carry both hydrophilic and lipophilic drugs [109]. This adaptability is due to their unique structure, typically comprising a lipid core that can encapsulate lipophilic drugs and an outer layer that can be modified to hold hydrophilic drugs [109]. This dual capability enables the co-delivery of different types of drugs within a single nanoparticle, making them ideal for complex treatment regimens. Furthermore, the lipid composition can be adjusted to achieve great encapsulation efficiency and high release rate of the drugs, ensuring that they are delivered in a controlled and targeted manner [110]. This versatility is particularly advantageous in developing treatments for conditions like alopecia, where precise and sustained drug delivery is essential for therapeutic efficiency.

### Biocompatibility and low toxicity

LNs are known for their biocompatibility and low toxicity, making them particularly suitable for treating alopecia [41]. These nanoparticles are typically composed of natural or synthetic lipids that closely resemble the lipids found in biological membrane [111], which allows them to be tolerated by the body. Their biocompatible nature means they can deliver therapeutic agents without generating significant immune responses or causing scalp irritation. This combination of biocompatibility and low toxicity ensures that lipid nanoparticles can safely and effectively deliver drugs to the target site, providing a promising approach for long term alopecia treatment.

## Challenges and limitations

While LNs present promising advancements in alopecia treatment, they also face several challenges and limitations. Drug loading and release profiles variability which can lead to inconsistent therapeutic outcomes is considered as a main drawback for this delivery system [112]. This variability may arise due to drugs encapsulation within the lipid matrix can differ, affecting the amount of drug delivered to the target site and the duration of its release. As reported for SLNs when used as topical nanocarriers is their flawless crystalline structure, which results in lesser drug loading and a stronger burst effect [113]. Additionally, the stability of lipid nanoparticles is a concern, as they can be sensitive to environmental factors such as temperature and humidity [114]. This sensitivity can result in changes to the physical and chemical properties of the nanoparticles over time, potentially reducing their effectiveness and shortening their shelf life.

Beyond formulation related limitations, the biological environment of the scalp poses additional challenges. Variability in sebum production, skin pH, and the presence of scalp microbiota can influence the penetration, retention, and performance of topically applied nanoformulations [115]. These variations pose challenges in achieving predictable and uniform response among patients.

Another significant limitation is the difficulty in scaling up production while maintaining the uniformity and quality of the nanoparticles. The precision required to produce LNs possessing consistent size, drug load, and release can be challenging to achieve on a larger industrial scale due to variation from batch to batch [116], which could limit their widespread availability and their use in clinical studies.

Additionally, the development and production of LNs can be costly and required multidisciplinary expertise. The sophisticated technology and precise processes required to create these formulations often results in higher production costs compared to traditional alopecia treatments. This increased cost may limit the accessibility of these advanced treatments for some patients.

Addressing these limitations, particularly issues related to production cost, scalability, and formulation stability, remains an important focus of ongoing research. Emerging formulation strategies and advanced design approaches may provide potential solutions to overcome these challenges in future developments.

### Future perspectives

Several emerging strategies recently appeared as promising candidates for enhancing lipid-based nanocarriers in alopecia treatment. Stimuli-responsive carriers, designed to release drugs in response to internal or external stimuli, such as pH, temperature, enzymes, redox conditions, light, magnetic fields, or ultrasound. By enabling site-specific and regulated medication release, these devices improve therapeutic results while reducing adverse effects. Their main benefits include greater solubility and stability for hydrophobic drugs, regulated their release to avoid premature leakage, and enhanced targeting efficiency by reacting only at disease locations. Their design can be customized using polymers, lipids, or inorganic materials to suit various applications and stimuli [117].

Moreover, by tackling the immune-mediated mechanisms causing hair follicle damage in AA, Janus kinase (JAK) inhibitors have become a promising therapy option [118]. JAK inhibitors avoid general immunosuppression while directly modulating the immunological processes causing hair follicle death in AA, in contrast to conventional non-specific AA therapies [119]. Numerous JAK inhibitor-loaded NLCs significantly improved hair follicle targeting, reversed hair loss in individuals with moderate to severe AA, and prevented systemic adverse effects [54, 120, 121].

In addition, advances in rational formulation design, including controlled particle size, surface modification, and optimization of lipid composition, may further improve follicular penetration, retention, and overall treatment efficiency. Exploring these approaches could address current limitations, such as stability and targeted delivery, and accelerate the clinical translation of lipid-based nanocarriers for alopecia therapy.

## Preclinical/clinical studies

Recognizing the potential of melatonin and antioxidant oils for androgenic alopecia, a study set out to create nanostructured lipid carriers (NLCs) designed for superior skin delivery and antioxidant benefits. The research involved a comprehensive characterization of the NLCs, including their nanometric size, negative charge, high drug entrapment, and sustained release over 6 h. They exhibited high antioxidant potential and good stability. Compared to a standard melatonin solution, the NLCs enhanced melatonin’s presence in the skin, increasing deposition 4.5-fold in the stratum corneum, sevenfold in the epidermis, and 6.8-fold in the dermis.

Clinical evaluation of the patients revealed that the melatonin NLCs provided more favorable results than the melatonin solution. This was evidenced by improvements in hair density and thickness, along with a reduction in hair loss. Therefore, this NLC delivery system represents a highly promising area in alopecia treatment [122].

Another study was focused on Coenzyme Q10 (CoQ10). Despite its anti-aging properties, CoQ10 faces challenges in therapeutic use due to its large molecular weight and poor water solubility hindering its absorption into the skin. Therefore, to overcome these limitations and improve CoQ10’s effectiveness in treating androgenic alopecia, this study explored different vesicular nanocarriers. The researchers formulated CoQ10 into liposomes, transfersomes, ethosomes, cerosomes, and transethosomes. Transethosomes emerged as the superior carrier, exhibiting a 146 nm particle size, a − 55 mV zeta potential, and a high entrapment efficiency of 97.63%. Also, transethosomes delivered over 95% of CoQ10 into the various skin layers. Clinical and dermoscopic examinations of patients with androgenic alopecia confirmed that CoQ10 delivered via transethosomes led to a significantly better clinical response than CoQ10 solution. This suggests that incorporating antioxidants like CoQ10 into this type of nanocarriers can greatly boost their therapeutic efficacy [123].

Briefly, alopecia poses significant psychological and social challenges, with existing treatments often limited by poor bioavailability and delivery challenges. Lipid-based nanocarriers have emerged as promising advanced drug delivery systems to resolve these issues.

Lipid-based nanocarriers have also been investigated in some patents concerned with the treatment of alopecia. An invention introduced a novel nanoliposome-microbubble conjugate designed to deliver hair loss drugs like finasteride, minoxidil, and dutasteride more effectively. While nanoliposomes can encapsulate drugs for improved delivery, they may struggle to penetrate the skin’s barrier effectively. Microbubbles, which are FDA-approved ultrasound contrast agents, can create temporary pores in cell membranes when exposed to ultrasound, enhancing drug entry. This invention a combined technology composed of hydrophobic gas-filled microbubbles chemically bound to nanoliposomes containing the drug. This conjugate aims to leverage the enhanced cellular delivery capability of microbubbles with the drug encapsulation benefits of nanoliposomes, leading to a more efficient and targeted treatment for hair loss by improving drug delivery into the dermal layer. The nanoliposomes were composed of lecithin, cholesterol and cationic phospholipids. While, the microbubbles contained amphoteric phospholipids, anionic phospholipids, cholesterol, cationic phospholipids, and lipids having disulfide groups [124]. In another patent, a liposomal topical formulation comprising dutasteride and embedded in an aqueous gel matrix comprising a gelling agent (a water-soluble silicone compound) was used and proved efficiency [125].

Some clinical trials investigated the potential enhancement of drug effect by nanoencapsulation. For example a study entitled ‘Role of Minoxidil in Alopecia Areata Transepidermal Drug Delivery of Minoxidil Via Either Fractional Carbon Dioxide Laser or Microneedling Versus Its Topical Nanoparticles Preparation for Treatment of Alopecia Areata’ (ClinicalTrials.gov ID NCT05587257), done in 2022 and sponsored by Assiut University compared three methods for treating alopecia areata: fractional CO2 laser or microneedling to enhance the penetration of 5% minoxidil, versus a minoxidil nanoparticle (Niosomes). https://clinicaltrials.gov/study/NCT05587257.

Another study entitled: Sildenafil-loaded Lipid-based Nanocarrier as a Potential Therapy for Alopecia Areata: A Randomized Clinical Study (ClinicalTrials.gov ID NCT06527729), was carried in Assiut University in 2024. It compared between 5% minoxidil gel and 1% sildenafil-loaded liposomes. https://clinicaltrials.gov/study/NCT06527729.

While preclinical results are promising, significant physiological differences in human scalp (e.g., hair density, sebum production, stratum corneum thickness) present hurdles for direct translation. Therefore, further research and clinical validation are needed.

## Regulatory landscape

The global Nanotechnology Drug Delivery Market is valued at USD 97.98 Billion in 2024 and is projected to reach USD 231.7 Billion by 2035 at a CAGR of 8.15%, indicating strong regulatory and commercial acceptance of nanotechnology-based delivery systems. Lipid-based systems are gaining popularity due to their safety profiles and regulatory acceptance [126].

Bringing nanotherapeutics to market faces significant hurdles including regulatory ambiguity [127]. While the U.S. Food and Drug Administration (FDA) lacks a formal definition for nanomaterials, it checks nanomaterials up to 1000 nm based on their size-dependent properties. The European Medicines Agency (EMA) defines pharmaceutical nanoparticles as nanoscale manufactured systems offering a clinical advantage. Both agencies agree on the necessity of comprehensive risk identification and management, demanding detailed characterization of nanoformulations (e.g., composition, size, morphology, stability) and a clear link between these attributes and the drug’s safety and effectiveness [128–130]. The assessment of product quality and safety must be performed. Also, understanding the interaction of nanoscale materials with biological systems, adequacy of testing approaches and evaluation of safety and effectiveness must be taken in consideration.

Manufacturing scale-up presents a major challenge, as maintaining consistent quality and physicochemical properties like particle size and morphology at an industrial level is difficult. Strategies to ensure stability and prevent agglomeration, such as using stabilizing agents are crucial. Also, another major hurdle in developing nanocarriers is the elevated cost of research and development (R&D). Creating these advanced drug delivery systems demands cutting-edge technology, specialized equipment, and extensive clinical trials, all of which necessitate substantial initial financial outlays.

In clinical trials, thorough biocompatibility and toxicity assessments are essential. It’s vital to understand the unique pharmacokinetic (PK) and pharmacodynamic (PD) profiles of drug delivery nanosystems, given their ability to improve bioavailability, bypass biological barriers, and control drug release. Finally, broader commercialization is hampered by the need to demonstrate bioequivalence for generic nanomedicines, the uncertainties surrounding these complex products, and the varying requirements between the FDA and EMA that complicate global development [127]. The FDA and EMA have accepted regulatory submissions for alopecia treatments, with decisions based on clinical data demonstrating significant scalp hair regrowth versus placebo and examination according to appropriate endpoints for demonstrating hair regrowth efficacy.

The safety evaluation of lipid-based nanocarriers for dermal application in alopecia treatment requires a multi-faceted approach, considering both general nanotoxicity principles and specific dermal considerations. The initial safety assessment begins with thorough physicochemical characterization. This includes precise determination of particle size, size distribution, zeta potential, morphology, drug loading efficiency, and release kinetics. Batch-to-batch consistency in these parameters is critical as any changes can influence biological interactions and toxicological profiles [131]. Then, in vitro biological assays comprising cytotoxicity study, inflammation and immunotoxicity, oxidative stress induced by nanocarriers, genotoxicity and mutagenicity are necessary [132, 133]. After these studies are performed successfully, ex vivo skin permeation and retention studies using excised human or animal skin models (e.g., Franz diffusion cells) are done [134–136], these studies quantify the amount of nanocarrier and encapsulated drug that penetrates and retains within different layers of the skin (stratum corneum, epidermis, dermis, hair follicles) versus systemic absorption. This is critical for assessing follicle targeting and minimizing systemic exposure, thereby reducing potential off-target effects.

In vivo dermal toxicity studies involving acute dermal irritation and sensitization in animal models are done to assess primary dermal irritation (erythema, edema) and delayed-type hypersensitivity reactions. Then, subchronic/chronic dermal toxicity repeated dose dermal toxicity studies evaluate the effects of prolonged exposure to nanocarriers on the skin and underlying tissues, as well as systemic absorption and accumulation in target organs [137, 138]. Histopathological examination of tissue biopsies is essential. Moreover, biocompatibility and biodistribution studies to assess the fate of nanocarriers in the body after dermal application, including their distribution to hair follicles, lymphatic system, and systemic circulation, as well as their degradation and clearance pathways are important.

Briefly, the regulatory pathway for nanotechnology-based pharmaceutical products is still evolving. Regulatory agencies like the FDA and EMA are developing necessary guidelines [129, 139]. Detailed characterization of the nanoparticles is required, including size distribution, shape, surface properties, charge (zeta potential), drug encapsulation efficiency, and release profile. Uniformity and consistency between batches is crucial. Also, preclinical studies are necessary to evaluate the safety profile of these formulations. This includes assessing potential local as well as systemic toxicity. Long-term safety studies are particularly important for chronic conditions like alopecia. In addition, preclinical and clinical trials are required to demonstrate the efficacy in promoting hair growth or reducing hair loss. These studies need to use appropriate animal models followed by well-designed human clinical trials. On the other hand, manufacturers must adhere to strict Good Manufacturing Practices (GMP) to ensure the quality and consistency of the final products. This includes controlling raw materials, manufacturing processes, and the final product. In addition, the potential environmental impact of nanoparticles must be taken in consideration.

Emphasizing the critical need for standardized human clinical protocols, the economic challenges associated with large-scale nano-manufacturing, and the current lack of specific and, streamlined regulatory pathways for topical nanomedicines remain great challenges.

## Conclusion

Alopecia remains a challenging condition to manage due to its multifactorial nature and conventional therapies limitations. Lipid-based nanoparticles hold significant promise for revolutionizing alopecia treatment by enabling more efficient, targeted and sustained delivery of therapeutic agents to hair follicles. However, overcoming formulation, manufacturing, and regulatory approval must be addressed is essential for their successful implementation. Continued research, including well designed preclinical and clinical studies, is essential to validate their safety and efficacy. With the right scientific and regulatory advancements, lipid based nanoparticles have the potential to be a prevalent treatment offering hope for individuals.

## Data Availability

No datasets were generated or analysed during the current study.

## Declarations

### Declaration of AI-assisted technologies in the writing process

AI-assisted technologies were only used to improve the readability and language of the manuscript. After use, the authors reviewed and edited the content as needed and take full responsibility for the content of the published article.

## References

[1] Rambwawasvika H, Dzomba P, Gwatidzo L. Alopecia types, current and future treatment. J Dermatol Cosmetol. 2021;5(4):93–9.

[2] Rahangdale PC, Wankhade AM. A review on-types and treatment of alopecia. Asian J Pharm Res. 2023;13(2):123–8.

[3] Schneider MR, Schmidt-Ullrich R, Paus R. The hair follicle as a dynamic miniorgan. Curr Biol. 2009;19(3):R132–42. 19211055 10.1016/j.cub.2008.12.005

[4] Noreen S, Pervaiz F, Maqbool I, Saleem M. Alopecia: review of epidemiology, pathogenesis, and novel treatment strategies.

[5] Houschyar KS, Borrelli MR, Tapking C, Popp D, Puladi B, Ooms M, et al. Molecular mechanisms of hair growth and regeneration: current understanding and novel paradigms. Dermatology. 2020;236(4):271–80. 32163945 10.1159/000506155

[6] Khairnar TD, Chavan GS, Sayyed MM, Gujarathi NA, Aher AA, Agrawal YO, et al. Recent trends in nanocarrier formulations of actives beyond minoxidil and 5-α reductase inhibitors in androgenetic alopecia management: a systematic review. J Drug Deliv Sci Technol. 2024. 10.1016/j.jddst.2024.105890.

[7] Alfutaimani AS, Alharbi NK, Alahmari SA, Alqabbani AA, Aldayel AM. Exploring the landscape of lipid nanoparticles (LNPs): a comprehensive review of LNPs types and biological sources of lipids. Int J Pharm X. 2024;8:100305. 39669003 10.1016/j.ijpx.2024.100305 PMC11635012

[8] Cardoso CO, Tolentino S, Gratieri T, Cunha-Filho M, Lopez RF, Gelfuso GM. Topical treatment for scarring and non-scarring alopecia: an overview of the current evidence. Clin Cosmet Investig Dermatol. 2021;14:485–99. 34012282 10.2147/CCID.S284435 PMC8126704

[9] Lee W-S, Lee H-J. Characteristics of androgenetic alopecia in Asian. Ann Dermatol. 2012;24(3):243. 22879706 10.5021/ad.2012.24.3.243 PMC3412231

[10] Islam N, Leung PS, Huntley AC, Gershwin ME. The autoimmune basis of alopecia areata: a comprehensive review. Autoimmun Rev. 2015;14(2):81–9. 25315746 10.1016/j.autrev.2014.10.014

[11] Alsantali A. Alopecia areata: a new treatment plan. Clin Cosmet Investig Dermatol. 2011;4:107–15. 21833161 10.2147/CCID.S22767 PMC3149478

[12] Ishida K, Ishida J, Kiyoko K. Psychosocial reaction patterns to alopecia in female patients with gynecological cancer undergoing chemotherapy. Asian Pac J Cancer Prev. 2015;16(3):1225–33. 25735360 10.7314/apjcp.2015.16.3.1225

[13] Keum DI, Pi L-Q, Hwang ST, Lee W-S. Protective effect of Korean Red Ginseng against chemotherapeutic drug-induced premature catagen development assessed with human hair follicle organ culture model. J Ginseng Res. 2016;40(2):169–75. 27158238 10.1016/j.jgr.2015.07.004 PMC4845051

[14] Ghias MH, Amin BD, Kutner AJ. Albendazole-induced anagen effluvium. JAAD Case Rep. 2020;6(1):54–6. 31909140 10.1016/j.jdcr.2019.08.010 PMC6938881

[15] Castelo-Soccio L. Diagnosis and management of alopecia in children. Pediatr Clin. 2014;61(2):427–42. 10.1016/j.pcl.2013.12.002 24636654

[16] Alves R, Grimalt R. Hair loss in children. Curr Probl Dermatol. 2015;47(3):55–66. 26370644 10.1159/000369405

[17] Grant JE, Chamberlain SR. Trichotillomania. Am J Psychiatry. 2016;173(9):868–74. 27581696 10.1176/appi.ajp.2016.15111432 PMC5328413

[18] Billero V, Miteva M. Traction alopecia: The root of the problem. Clin Cosmet Investig Dermatol. 2018. 10.2147/ccid.s137296. 29670386 10.2147/CCID.S137296 PMC5896661

[19] Mesrati H, Amouri M, Chaaben H, Masmoudi A, Boudaya S, Turki H. Aplasia cutis congenita: report of 22 cases. Int J Dermatol. 2015;54(12):1370–5. 26016611 10.1111/ijd.12707

[20] Maekawa M, Ohnishi T, Balan S, Hisano Y, Nozaki Y, Ohba H, et al. Thiosulfate promotes hair growth in mouse model. Biosci Biotechnol Biochem. 2019;83(1):114–22. 30200826 10.1080/09168451.2018.1518705

[21] Ong MM, Li Y, Lipner SR. Oral Minoxidil for alopecia treatment: risks, benefits, and recommendations. Am J Clin Dermatol. 2025;27:101–19. 41118052 10.1007/s40257-025-00990-4

[22] Kelly Y, Blanco A, Tosti A. Androgenetic alopecia: an update of treatment options. Drugs. 2016;76:1349–64. 27554257 10.1007/s40265-016-0629-5

[23] Allam AT, El-Shiekh RA, El-Dessouki AM, Gamil NM, Eisa NM, Ayoub MM, et al. Pathophysiology, conventional treatments, and evidence-based herbal remedies of hair loss with a systematic review of controlled clinical trials. Naunyn-Schmiedeberg’s Arch Pharmacol. 2025;398:16311–44. 40536553 10.1007/s00210-025-04286-6 PMC12678491

[24] Devjani S, Ezemma O, Kelley KJ, Stratton E, Senna M. Androgenetic alopecia: therapy update. Drugs. 2023;83(8):701–15. 37166619 10.1007/s40265-023-01880-x PMC10173235

[25] Freitas E, Guttman-Yassky E, Torres T. Baricitinib for the treatment of alopecia areata. Drugs. 2023;83(9):761–70. 37195491 10.1007/s40265-023-01873-w PMC10191081

[26] Wada‐Irimada M, Takahashi T, Sekine M, Okazaki T, Takahashi T, Chiba T, et al. Predictive factors for treatment responses to baricitinib in severe alopecia areata: a retrospective, multivariate analysis of 70 cases from a single center. J Dermatol. 2025;52(4):701–11. 39868612 10.1111/1346-8138.17641

[27] Shirley M. Ritlecitinib in severe alopecia areata: a profile of its use. Drugs Ther Perspect. 2024;40(9):341–9.

[28] Piliang M, Lynde C, King B, Mirmirani P, Sinclair R, Senna M, et al. Sustained hair regrowth with continued ritlecitinib treatment through week 48 in patients with Alopecia Areata with or without early target responses: post hoc analysis of the ALLEGRO phase 2b/3 trial. J Am Acad Dermatol. 2025;92(2):276–84. 39423930 10.1016/j.jaad.2024.09.064

[29] Javed T. FDA approval of Deuruxolitinib: a new treatment option for severe Alopecia Areata. Clin Res. 2024;5(1):1–3.

[30] King B, Senna MM, Mesinkovska NA, Lynde C, Zirwas M, Maari C, et al. Efficacy and safety of deuruxolitinib, an oral selective Janus kinase inhibitor, in adults with alopecia areata: results from the Phase 3 randomized, controlled trial (THRIVE-AA1). J Am Acad Dermatol. 2024;91(5):880–8. 39053611 10.1016/j.jaad.2024.06.097

[31] Zgonc Škulj A, Poljšak N, Kočevar Glavač N, Kreft S. Herbal preparations for the treatment of hair loss. Arch Dermatol Res. 2020;312:395–406. 31680216 10.1007/s00403-019-02003-x

[32] Damodaran RG, Gupta R. Hair loss and the applied techniques for identification of novel hair growth promoters for hair re-growth. Pharmacogn J. 2011;3(22):1–5.

[33] Strazzulla LC, Wang EHC, Avila L, Sicco KL, Brinster N, Christiano AM, et al. Alopecia areata: an appraisal of new treatment approaches and overview of current therapies. J Am Acad Dermatol. 2018;78(1):15–24. 29241773 10.1016/j.jaad.2017.04.1142

[34] Pena-Rodríguez E, García-Vega L, Lajarin Reinares M, Pastor-Anglada M, Pérez-Torras S, Fernandez-Campos F. Latanoprost-loaded Nanotransfersomes designed for scalp administration enhance keratinocytes proliferation. Mol Pharm. 2022;20(5):2317–25. 36503244 10.1021/acs.molpharmaceut.2c00796 PMC10155202

[35] de Oliveira TA, Marchesan LB, Spritzer PM. Potassium levels in women with Polycystic Ovary Syndrome using spironolactone for long‐term. Clin Endocrinol Oxf. 2024;100(3):278–83. 38127445 10.1111/cen.15008

[36] Iszczuk OK, Silldorff J, Fura T, Dudek M, Zaucha R, Gajkiewicz M, et al. Current state of knowledge about spironolactone-induded gynecomastia. Review 2024. Qual Sport. 2024;17:53767.

[37] Perez SM, Nguyen B, Senna MM. Efficacy and safety of bicalutamide in female hair loss: a review of the literature. JAAD Rev. 2025;4:61–8.

[38] Lim SY, Bolster MB. Corticosteroids. In: Neurorheumatology: a comprehenisve guide to immune mediated disorders of the nervous system. Springer; 2019. p. 261–7.

[39] Costa C, Cavaco-Paulo A, Matamá T. Mapping hair follicle-targeted delivery by particle systems: What has science accomplished so far? Int J Pharm. 2021;610:121273. 34763036 10.1016/j.ijpharm.2021.121273

[40] Shaikh ZSA, Patel BAA, Patil SG, Maniyar ARS. Nanotechnology-based strategies for hair follicle regeneration in androgenetic alopecia. Mater Proc. 2023;14(1):57.

[41] Oliveira PM, Alencar-Silva T, Pires FQ, Cunha-Filho M, Gratieri T, Carvalho JL, et al. Nanostructured lipid carriers loaded with an association of minoxidil and latanoprost for targeted topical therapy of alopecia. Eur J Pharm Biopharm. 2022;172:78–88. 35143972 10.1016/j.ejpb.2022.02.003

[42] Gomes MJ, Martins S, Ferreira D, Segundo MA, Reis S. Lipid nanoparticles for topical and transdermal application for alopecia treatment: development, physicochemical characterization, and in vitro release and penetration studies. Int J Nanomedicine. 2014;9:1231–42. 24634584 10.2147/IJN.S45561 PMC3952901

[43] Trucillo P. Biomaterials for drug delivery and human applications. Materials. 2024;17(2):456. 38255624 10.3390/ma17020456 PMC10817481

[44] Khairnar TD, Chavan GS, Sayyed MM, Gujarathi NA, Aher AA, Agrawal YO, et al. Recent trends in nanocarrier formulations of actives beyond minoxidil and 5-α reductase inhibitors in androgenetic alopecia management: a systematic review. J Drug Deliv Sci Technol. 2024;98:105890.

[45] Paliwal R, Paliwal SR, Kenwat R, Kurmi BD, Sahu MK. Solid lipid nanoparticles: a review on recent perspectives and patents. Expert Opin Ther Pat. 2020;30(3):179–94. 32003260 10.1080/13543776.2020.1720649

[46] Wosicka-Frąckowiak H, Cal K, Stefanowska J, Główka E, Nowacka M, Struck-Lewicka W, et al. Roxithromycin-loaded lipid nanoparticles for follicular targeting. Int J Pharm. 2015;495(2):807–15. 26456292 10.1016/j.ijpharm.2015.09.068

[47] Hamishehkar H, Ghanbarzadeh S, Sepehran S, Javadzadeh Y, Adib ZM, Kouhsoltani M. Histological assessment of follicular delivery of flutamide by solid lipid nanoparticles: potential tool for the treatment of androgenic alopecia. Drug Dev Ind Pharm. 2016;42(6):846–53. 26154267 10.3109/03639045.2015.1062896

[48] Khan S, Sharma A, Jain V. An overview of nanostructured lipid carriers and its application in drug delivery through different routes. Adv Pharm Bull. 2023;13(3):446–60. 37646052 10.34172/apb.2023.056 PMC10460807

[49] Lin YK, Al-Suwayeh SA, Leu YL, Shen FM, Fang JY. Squalene-containing nanostructured lipid carriers promote percutaneous absorption and hair follicle targeting of diphencyprone for treating alopecia areata. Pharm Res. 2013;30(2):435–46. 23070602 10.1007/s11095-012-0888-0

[50] Kushwaha P, Usmani S, Sufiyan M, Singh P. Innovating alopecia treatment: Nanostructured lipid carriers as advanced delivery platforms. Naunyn-Schmiedebergs Arch Pharmacol. 2025. 10.1007/s00210-025-03784-x. 39825967 10.1007/s00210-025-03784-x

[51] Wang W, Chen L, Huang X, Shao A. Preparation and characterization of minoxidil loaded nanostructured lipid carriers. AAPS PharmSciTech. 2017;18(2):509–16. 27120090 10.1208/s12249-016-0519-x

[52] Aljuffali IA, Pan TL, Sung CT, Chang SH, Fang JY. Anti-PDGF receptor β antibody-conjugated squarticles loaded with minoxidil for alopecia treatment by targeting hair follicles and dermal papilla cells. Nanomedicine. 2015;11(6):1321–30. 25933696 10.1016/j.nano.2015.04.009

[53] Shamma RN, Aburahma MH. Follicular delivery of spironolactone via nanostructured lipid carriers for management of alopecia. Int J Nanomed. 2014;9:5449–60. 10.2147/IJN.S73010 PMC4251754 25473283

[54] Li Q, Wang Y, Guo Q, Cao J, Feng Y, Ke X. Nanostructured lipid carriers promote percutaneous absorption and hair follicle targeting of tofacitinib for treating alopecia areata. J Control Release. 2024;372:778–94. 38936744 10.1016/j.jconrel.2024.06.060

[55] Ananth P, Koland M. Topical delivery of fenugreek seed extract loaded solid lipid nanoparticles based hydrogels for alopecia. J Pharm Res Int. 2021;33:231–41.

[56] Prabahar K, Udhumansha U, Elsherbiny N, Qushawy M. Microneedle mediated transdermal delivery of β-sitosterol loaded nanostructured lipid nanoparticles for androgenic alopecia. Drug Deliv. 2022;29(1):3022–34. 36110028 10.1080/10717544.2022.2120927 PMC10003132

[57] Hariyanti H, Mauludin R, Sumirtapura YC, Kurniati NF. Activity and safety of cinchonine nanostructured lipid carriers as a hair growth stimulant in mice model of androgenetic alopecia. Sains Malaysiana. 2023;52(6):1671–83.

[58] Daneshmand S, Niazi M, Fazeli-Nasab B, Asili J, Golmohammadzadeh S, Sayyed R. Solid lipid nanoparticles of *Platycladus orientalis* L. possessing 5-alpha reductase inhibiting activity for treating hair loss and hirsutism. J Med Plants By-Prod. 2023;13(1):233–46.

[59] Akbarzadeh A, Rezaei-Sadabady R, Davaran S, Joo SW, Zarghami N, Hanifehpour Y, et al. Liposome: classification, preparation, and applications. Nanoscale Res Lett. 2013;8(1):102. 23432972 10.1186/1556-276X-8-102 PMC3599573

[60] AbouSamra MM. Liposomal nano-carriers mediated targeting of liver disorders: mechanisms and applications. J Liposome Res. 2024;34:728–43. 38988127 10.1080/08982104.2024.2377085

[61] Ahmadi Ashtiani HR, Bishe P, Lashgari N, Nilforoushzadeh MA, Zare S. Liposomes in cosmetics. J Skin Stem Cell. 2016;3(3):e65815.

[62] Dymek M, Sikora E. Liposomes as biocompatible and smart delivery systems—the current state. Adv Colloid Interface Sci. 2022;309:102757. 36152374 10.1016/j.cis.2022.102757

[63] Geusens B, Strobbe T, Bracke S, Dynoodt P, Sanders N, Van Gele M, et al. Lipid-mediated gene delivery to the skin. Eur J Pharm Sci. 2011;43(4):199–211. 21515366 10.1016/j.ejps.2011.04.003

[64] Cosco D, Celia C, Cilurzo F, Trapasso E, Paolino D. Colloidal carriers for the enhanced delivery through the skin. Expert Opin Drug Deliv. 2008;5(7):737–55. 18590459 10.1517/17425247.5.7.737

[65] Xu H-L, Chen P-P, Wang L-f, Xue W, Fu T-L. Hair regenerative effect of silk fibroin hydrogel with incorporation of FGF-2-liposome and its potential mechanism in mice with testosterone-induced alopecia areata. J Drug Deliv Sci Technol. 2018;48:128–36.

[66] Hooper D, Kraemer W, Focht B, Volek J, DuPont W, Caldwell L, et al. Endocrinological roles for testosterone in resistance exercise responses and adaptations. Sports Med. 2017;47:1709–20. 28224307 10.1007/s40279-017-0698-y

[67] Grymowicz M, Rudnicka E, Podfigurna A, Napierala P, Smolarczyk R, Smolarczyk K, et al. Hormonal effects on hair follicles. Int J Mol Sci. 2020;21(15):5342. 32731328 10.3390/ijms21155342 PMC7432488

[68] Zhang X, Li S, Dong Y, Rong H, Zhao J, Hu H. A multifunctional cholesterol-free liposomal platform based on protopanaxadiol for alopecia therapy. Nano Res. 2022;15(10):9498–510.

[69] Xing H, Peng H, Yang Y, Lv K, Zhou S, Pan X, et al. Nitric oxide synergizes minoxidil delivered by transdermal hyaluronic acid liposomes for multimodal androgenetic-alopecia therapy. Bioact Mater. 2024;32:190–205. 37859688 10.1016/j.bioactmat.2023.09.021 PMC10582348

[70] Rajan R, Jose S, Mukund VP, Vasudevan DT. Transfersomes—a vesicular transdermal delivery system for enhanced drug permeation. J Adv Pharm Technol Res. 2011;2(3):138–43. 22171309 10.4103/2231-4040.85524 PMC3217704

[71] Cevc G, Blume G. New, highly efficient formulation of diclofenac for the topical, transdermal administration in ultradeformable drug carriers, Transfersomes. Biochimica et Biophysica Acta (BBA). 2001;1514(2):191–205. 11557020 10.1016/s0005-2736(01)00369-8

[72] Zhang ZJ, Michniak-Kohn B. Flavosomes, novel deformable liposomes for the co-delivery of anti-inflammatory compounds to skin. Int J Pharm. 2020;585:119500. 32512226 10.1016/j.ijpharm.2020.119500

[73] Opatha SAT, Titapiwatanakun V, Chutoprapat R. Transfersomes: a promising nanoencapsulation technique for transdermal drug delivery. Pharmaceutics. 2020;12(9):855. 32916782 10.3390/pharmaceutics12090855 PMC7559928

[74] Rai S, Pandey V, Rai G. Transfersomes as versatile and flexible nano-vesicular carriers in skin cancer therapy: the state of the art. Nano Rev Exp. 2017;8(1):1325708. 30410704 10.1080/20022727.2017.1325708 PMC6167026

[75] Ahmed OA, Rizq WY. Finasteride nano-transferosomal gel formula for management of androgenetic alopecia: ex vivo investigational approach. Drug Des Devel Ther. 2018;12:2259–65. 30104862 10.2147/DDDT.S171888 PMC6070339

[76] Yan A, Ruan R, Zhu X, Qiang W, Guan Y, Yu Q, et al. Co-delivery of minoxidil and tocopherol acetate ethosomes to reshape the hair follicular microenvironment and promote hair regeneration in androgenetic alopecia. Int J Pharm. 2023;646:123498. 37820942 10.1016/j.ijpharm.2023.123498

[77] Liu X, Kong X, Xu L, Su Y, Xu S, Pang X, et al. Synergistic therapeutic effect of ginsenoside Rg3 modified minoxidil transfersomes (MXD-Rg3@TFs) on androgenic alopecia in C57BL/6 mice. Int J Pharm. 2024;654:123963. 38430952 10.1016/j.ijpharm.2024.123963

[78] Liu X, Guo F, Liang D, Li Z, Cao Y, Chen M, et al. Development and evaluation of finasteride niosomes targeting to hair follicles for the management of androgenic alopecia. J Drug Deliv Sci Technol. 2023;86:104725.

[79] Rungseevijitprapa W, Wichayapreechar P, Sivamaruthi BS, Jinarat D, Chaiyasut C. Optimization and transfollicular delivery of finasteride-loaded proniosomes for hair growth stimulation in C57BL/6Mlac mice. Pharmaceutics. 2021. 10.3390/pharmaceutics13122177. 34959458 10.3390/pharmaceutics13122177 PMC8706991

[80] Rani D, Sharma V, Manchanda R, Chaurasia H. Formulation, design and optimization of glycerosomes for topical delivery of minoxidil. Res J Pharm Technol. 2021;14:2367–74.

[81] Allam AA, Fathalla D, Safwat MA, Soliman GM. Transferosomes versus transethosomes for the dermal delivery for minoxidil: preparation and in vitro/ex vivo appraisal. J Drug Deliv Sci Technol. 2022;76:103790.

[82] Salim S, Kamalasanan K. Controlled drug delivery for alopecia: a review. J Control Release. 2020;325:84–99. 32619746 10.1016/j.jconrel.2020.06.019

[83] Jaiswal M, Dudhe R, Sharma PK. Nanoemulsion: an advanced mode of drug delivery system. 3 Biotech. 2015;5(2):123–7. 28324579 10.1007/s13205-014-0214-0 PMC4362737

[84] Preeti A, Sambhakar S, Malik R, Bhatia S, Al Harrasi A, Rani C, et al. Nanoemulsion: an emerging novel technology for improving the bioavailability of drugs. Scientifica. 2023;2023:6640103. 37928749 10.1155/2023/6640103 PMC10625491

[85] Mushtaq A, Mohd Wani S, Malik AR, Gull A, Ramniwas S, Ahmad Nayik G, et al. Recent insights into nanoemulsions: their preparation, properties and applications. Food Chem X. 2023;18:100684. 37131847 10.1016/j.fochx.2023.100684 PMC10149285

[86] Cardoso SA, Barradas TN. Developing formulations for drug follicular targeting: nanoemulsions loaded with minoxidil and clove oil. J Drug Deliv Sci Technol. 2020;59:101908.

[87] Escamilla-Cruz M, Magaña M, Escandón-Perez S, Bello-Chavolla OY. Use of 5-alpha reductase inhibitors in dermatology: a narrative review. Dermatol Therapy. 2023;13(8):1721–31. 10.1007/s13555-023-00974-4 PMC10366043 37432644

[88] Memar Bashi Aval M, Hoveizi E, Mombeiny R, Kazemi M, Saeedi S, Tavakol S. Dutasteride nanoemulsion preparation to inhibit 5-alpha-hair follicle reductase enzymes in the hair follicle; an ex vivo study. IET Nanobiotechnol. 2023;17(1):13–21. 36314605 10.1049/nbt2.12101 PMC9932434

[89] Palakkal S, Cortial A, Frušić-Zlotkin M, Soroka Y, Tzur T, Nassar T, et al. Effect of cyclosporine A—tempol topical gel for the treatment of alopecia and anti-inflammatory disorders. Int J Pharm. 2023;642:123121. 37307961 10.1016/j.ijpharm.2023.123121

[90] Maged A, Kamel R, Mahmoud AA, Elkhawas YA, Mohamed SK, Khalil N. Sildenafil-chitosan nanocomplexes in rosemary-infused smart emulgel: sustainable drug delivery with improved hair regeneration efficacy. J Drug Deliv Sci Technol. 2025;114:107502.

[91] Erdoğar N, Gür B, Örgül D. Recent developments of novel nanotechnology-based drug delivery systems for dermal and transdermal applications. Eur J Pharm Sci. 2026;217:107413. 41389911 10.1016/j.ejps.2025.107413

[92] Yang G, Chen Q, Wen D, Chen Z, Wang J, Chen G, et al. A Therapeutic microneedle patch made from hair-derived keratin for promoting hair regrowth. ACS Nano. 2019;13(4):4354–60. 30942567 10.1021/acsnano.8b09573

[93] Randolph M, Tosti A. Oral minoxidil treatment for hair loss: a review of efficacy and safety. J Am Acad Dermatol. 2021;84(3):737–46. 32622136 10.1016/j.jaad.2020.06.1009

[94] Habeshian KA, Cohen BA. Current issues in the treatment of acne vulgaris. Pediatrics. 2020;145(Suppl 2):S225–30. 32358215 10.1542/peds.2019-2056L

[95] Garg V, Nirmal J, Riadi Y, Kesharwani P, Kohli K, Jain GK. Amelioration of endotoxin-induced uveitis in rabbit by topical administration of tacrolimus Proglycosome nano-vesicles. J Pharm Sci. 2021;110(2):871–5. 33157078 10.1016/j.xphs.2020.10.060

[96] Mishra P, Handa M, Ujjwal RR, Singh V, Kesharwani P, Shukla R. Potential of nanoparticulate based delivery systems for effective management of alopecia. Colloids Surf B Biointerfaces. 2021;208:112050. 34418723 10.1016/j.colsurfb.2021.112050

[97] Pardeike J, Hommoss A, Müller RH. Lipid nanoparticles (SLN, NLC) in cosmetic and pharmaceutical dermal products. Int J Pharm. 2009;366(1–2):170–84. 18992314 10.1016/j.ijpharm.2008.10.003

[98] Kwon TK, Kim JC. In vitro skin permeation of monoolein nanoparticles containing hydroxypropyl beta-cyclodextrin/minoxidil complex. Int J Pharm. 2010;392(1–2):268–73. 20362653 10.1016/j.ijpharm.2010.03.049

[99] Souto EB, Müller RH. Cosmetic features and applications of lipid nanoparticles (SLN, NLC). Int J Cosmet Sci. 2008;30(3):157–65. 18452432 10.1111/j.1468-2494.2008.00433.x

[100] Nayak AP, Tiyaboonchai W, Patankar S, Madhusudhan B, Souto EB. Curcuminoids-loaded lipid nanoparticles: novel approach towards malaria treatment. Colloids Surf B Biointerfaces. 2010;81(1):263–73. 20688493 10.1016/j.colsurfb.2010.07.020

[101] Mehnert W, Mäder K. Solid lipid nanoparticles: production, characterization and applications. Adv Drug Deliv Rev. 2001;47(2):165–96. 11311991 10.1016/s0169-409x(01)00105-3

[102] AbouSamra MM, Mohsen AM. Solid lipid nanoparticles and nanostructured lipid carriers of tolnaftate: design, optimization and in-vitro evaluation. Int J Pharm Pharm Sci. 2016;8(1):380–5.

[103] Jyothi VGS, Ghouse SM, Khatri DK, Nanduri S, Singh SB, Madan J. Lipid nanoparticles in topical dermal drug delivery: Does chemistry of lipid persuade skin penetration? J Drug Deliv Sci Technol. 2022;69:103176.

[104] Hallan SS, Sguizzato M, Esposito E, Cortesi R. Challenges in the physical characterization of lipid nanoparticles. Pharmaceutics. 2021. 10.3390/pharmaceutics13040549. 33919859 10.3390/pharmaceutics13040549 PMC8070758

[105] Wissing SA, Müller RH. The influence of solid lipid nanoparticles on skin hydration and viscoelasticity—in vivo study. Eur J Pharm Biopharm. 2003;56(1):67–72. 12837483 10.1016/s0939-6411(03)00040-7

[106] Arora R, Katiyar SS, Kushwah V, Jain S. Solid lipid nanoparticles and nanostructured lipid carrier-based nanotherapeutics in treatment of psoriasis: a comparative study. Expert Opin Drug Deliv. 2017;14(2):165–77. 27882780 10.1080/17425247.2017.1264386

[107] Garcês A, Amaral MH, Sousa Lobo JM, Silva AC. Formulations based on solid lipid nanoparticles (SLN) and nanostructured lipid carriers (NLC) for cutaneous use: a review. Eur J Pharm Sci. 2018;112:159–67. 29183800 10.1016/j.ejps.2017.11.023

[108] Lademann J, Richter H, Teichmann A, Otberg N, Blume-Peytavi U, Luengo J, et al. Nanoparticles--an efficient carrier for drug delivery into the hair follicles. Eur J Pharm Biopharm. 2007;66(2):159–64. 17169540 10.1016/j.ejpb.2006.10.019

[109] Seo Y, Lim H, Park H, Yu J, An J, Yoo HY, et al. Recent progress of lipid nanoparticles-based lipophilic drug delivery: focus on surface modifications. Pharmaceutics. 2023;15(3):772. 36986633 10.3390/pharmaceutics15030772 PMC10058399

[110] Rajeeva BB, Menz R, Zheng Y. Towards rational design of multifunctional theranostic nanoparticles: What barriers do we need to overcome? Taylor & Francis; 2014. p. 1767–70. 10.2217/nnm.14.103 25325238

[111] Puri A, Loomis K, Smith B, Lee JH, Yavlovich A, Heldman E, et al. Lipid-based nanoparticles as pharmaceutical drug carriers: from concepts to clinic. Crit Rev Ther Drug Carrier Syst. 2009;26(6):523–80. 20402623 10.1615/critrevtherdrugcarriersyst.v26.i6.10 PMC2885142

[112] Mehta M, Bui TA, Yang X, Aksoy Y, Goldys EM, Deng W. Lipid-based nanoparticles for drug/gene delivery: an overview of the production techniques and difficulties encountered in their industrial development. ACS Mater Au. 2023;3(6):600–19. 38089666 10.1021/acsmaterialsau.3c00032 PMC10636777

[113] Chantaburanan T, Teeranachaideekul V, Chantasart D, Jintapattanakit A, Junyaprasert VB. Effect of binary solid lipid matrix of wax and triglyceride on lipid crystallinity, drug-lipid interaction and drug release of ibuprofen-loaded solid lipid nanoparticles (SLN) for dermal delivery. J Colloid Interface Sci. 2017;504:247–56. 28551519 10.1016/j.jcis.2017.05.038

[114] Ashfaq R, Rasul A, Asghar S, Kovács A, Berkó S, Budai-Szűcs M. Lipid nanoparticles: an effective tool to improve the bioavailability of nutraceuticals. Int J Mol Sci. 2023. 10.3390/ijms242115764. 37958750 10.3390/ijms242115764 PMC10648376

[115] Paul JK, Azmal M, Talukder OF, Haque ANMSNB, Meem M, Ghosh A. Unlocking the secrets of the hair microbiome: from scalp health to therapeutic advances. Microbe. 2025;7:100353.

[116] Xu L, Wang X, Liu Y, Yang G, Falconer RJ, Zhao C-X. Lipid nanoparticles for drug delivery. Adv NanoBiomed Res. 2022;2(2):2100109.

[117] Vikhe SR, Pathade PM, Tambe BR. Stimuli-responsive nanocarriers: revolutionizing site-specific drug release. Med Pharm J. 2025. 10.55940/medphar2025118.

[118] Sanchez K, Englander H, Salloum L, Gregoire S, Biba U, Ershadi S, et al. Evaluating current and emergent JAK inhibitors for alopecia areata: a narrative review. Dermatol Ther (Heidelb). 2025;15(10):2749–64. 40794245 10.1007/s13555-025-01517-9 PMC12454744

[119] Malhotra K, Madke B. An updated review on current treatment of alopecia areata and newer therapeutic options. Int J Trichology. 2023;15(1):3–12. 37305188 10.4103/ijt.ijt_28_21 PMC10251289

[120] Zheng L, Du Y, Zhang L, Jin F, Li W, Zhou X, et al. Enhanced therapeutic effects of all-trans retinoic acid nanostructured lipid carrier composite gel drug delivery system for alopecia areata. J Nanobiotechnol. 2025;23(1):351. 10.1186/s12951-025-03407-w PMC12083027 40380336

[121] Kim J, Song SY, Sung JH. Recent advances in drug development for hair loss. Int J Mol Sci. 2025. 10.3390/ijms26083461. 40331976 10.3390/ijms26083461 PMC12026576

[122] Hatem S, Nasr M, Moftah NH, Ragai MH, Geneidi AS, Elkheshen SA. Clinical cosmeceutical repurposing of melatonin in androgenic alopecia using nanostructured lipid carriers prepared with antioxidant oils. Expert Opin Drug Deliv. 2018;15(10):927–35. 30169980 10.1080/17425247.2018.1517740

[123] El-Zaafarany GM, Abdel-Aziz RT, Montaser MHA, Nasr M. Coenzyme Q10 phospholipidic vesicular formulations for treatment of androgenic alopecia: ex vivo permeation and clinical appraisal. Expert Opin Drug Deliv. 2021;18(10):1513–22. 34047661 10.1080/17425247.2021.1936497

[124] ユ, キョンナム, inventor. Nanoliposomes-microbubble conjugates containing therapeutic agents for hair loss 2021.

[125] Victor H, Mauro C, inventors. Topical formulations comprising dutasteride for treating dermatological disorders including male pattern baldness, 2021.

[126] Nanotechnology drug delivery market: a deep-dive analysis [Internet]. 2025.

[127] Kamel R, Nocca G, Mazzinelli E, Abd El-Karim SS, Papa V, Cacciotti I, et al. Unveiling the potential of nanoclays in pharmaceuticals. AAPS PharmSciTech. 2025;26(5):167. 40495098 10.1208/s12249-025-03157-w

[128] Rodríguez-Gómez FD, Monferrer D, Penon O, Rivera-Gil P. Regulatory pathways and guidelines for nanotechnology-enabled health products: a comparative review of EU and US frameworks. Front Med. 2025;12:1544393. 10.3389/fmed.2025.1544393 PMC11919859 40109724

[129] Oualikene-Gonin W, Sautou V, Ezan E, Bastos H, Bellissant E, Belgodère L, et al. Regulatory assessment of nano-enabled health products in public health interest. Position of the scientific advisory board of the French National Agency for the Safety of Medicines and Health Products. Front Public Health. 2023;11:1125577. 36935690 10.3389/fpubh.2023.1125577 PMC10018019

[130] D’Mello SR, Cruz CN, Chen ML, Kapoor M, Lee SL, Tyner KM. The evolving landscape of drug products containing nanomaterials in the United States. Nat Nanotechnol. 2017;12(6):523–9. 28436961 10.1038/nnano.2017.67

[131] Nel AE, Mädler L, Velegol D, Xia T, Hoek EM, Somasundaran P, et al. Understanding biophysicochemical interactions at the nano–bio interface. Nat Mater. 2009;8(7):543–57. 19525947 10.1038/nmat2442

[132] Panghal A, Flora SJS. Toxicity evaluation of nanomedicine. In: Recent advances in therapeutic drug monitoring and clinical toxicology. Springer; 2022. p. 323–45.

[133] Saifi MA, Khan W, Godugu C. Cytotoxicity of nanomaterials: using nanotoxicology to address the safety concerns of nanoparticles. Pharm Nanotechnol. 2018;6(1):3–16. 29065848 10.2174/2211738505666171023152928

[134] Abdel Fadeel DA, Kamel R, Fadel M. PEGylated lipid nanocarrier for enhancing photodynamic therapy of skin carcinoma using curcumin: in-vitro/in-vivo studies and histopathological examination. Sci Rep. 2020;10(1):10435. 32591621 10.1038/s41598-020-67349-z PMC7320133

[135] Kamel R, Abbas H. Self-assembled carbohydrate hydrogels for prolonged pain management. Pharm Dev Technol. 2013;18(5):990–1004. 21950732 10.3109/10837450.2011.609992

[136] Hadgraft J, Lane ME. Skin permeation: the years of enlightenment. Int J Pharm. 2005;305(1–2):2–12. 16246513 10.1016/j.ijpharm.2005.07.014

[137] Crosera M, Bovenzi M, Maina G, Adami G, Zanette C, Florio C, et al. Nanoparticle dermal absorption and toxicity: a review of the literature. Int Arch Occup Environ Health. 2009;82(9):1043–55. 19705142 10.1007/s00420-009-0458-x

[138] Puglia C, Blasi P, Rizza L, Schoubben A, Bonina F, Rossi C, et al. Lipid nanoparticles for prolonged topical delivery: an in vitro and in vivo investigation. Int J Pharm. 2008;357(1–2):295–304. 18343059 10.1016/j.ijpharm.2008.01.045

[139] Rahman Z, Charoo NA, Akhter S, Beg S, Reddy IK, Khan MA. Nanotechnology-based drug products: science and regulatory considerations. In: Nanoscale fabrication, optimization, scale-up and biological aspects of pharmaceutical nanotechnology. Elsevier; 2018. p. 619–55.

